# Sustainability of the Effects and Impacts of Using Digital Technology to Extend Maternal Health Services to Rural and Hard-to-Reach Populations: Experience From Southwest Nigeria

**DOI:** 10.3389/fgwh.2022.696529

**Published:** 2022-02-08

**Authors:** David Akeju, Babasola Okusanya, Kehinde Okunade, Adegbenga Ajepe, Matthew J. Allsop, Bassey Ebenso

**Affiliations:** ^1^Department of Sociology, University of Lagos, Lagos, Nigeria; ^2^Department of Obstetrics and Gynaecology, College of Medicine, University of Lagos, Lagos, Nigeria; ^3^Department of Obstetrics and Gynaecology, Lagos University Teaching Hospital, Lagos, Nigeria; ^4^Academic Unit of Palliative Care, University of Leeds, Leeds, United Kingdom; ^5^Nuffield Centre for International Health and Development, University of Leeds, Leeds, United Kingdom

**Keywords:** evaluation of digital technology, most significant change, underserved population, maternal health service, Nigeria, sustainability of impacts

## Abstract

**Background:**

Nigeria has one of the worst health and development profiles globally. A weak health system, poor infrastructure, and varied socio-cultural factors are cited as inhibitors to optimal health system performance and improved maternal and child health status. eHealth has become a major solution to closing these gaps in health care delivery in low- and middle-income countries (LMICs). This research reports the use of satellite communication (SatCom) technology and the existing 3G mobile network for providing video training (VTR) for health workers and improving the digitization of healthcare data.

**Objective:**

To evaluate whether the expected project outcomes that were achieved at the end-line evaluation of 2019 were sustained 12 months after the project ended.

**Methods:**

From March 2017 to March 2019, digital innovations including VTR and data digitization interventions were delivered in 62 healthcare facilities in Ondo State, southwest Nigeria, most of which lacked access to a 3G mobile network. Data collection for the evaluation combined documents' review with quantitative data extracted from health facility registers, and 24 of the most significant change stories to assess the longevity of the outcomes and impacts of digital innovation in the four domains of healthcare: use of eHealth technology for data management, utilization of health facilities by patients, the standard of care, and staff attitude. Stories of the most significant changes were audio-recorded, transcribed for analysis, and categorized by the above domains to identify the most significant changes 12 months after the project closedown.

**Results:**

Findings showed that four project outcomes which were achieved at end-line evaluation were sustained 12 months after project closedown namely: staff motivation and satisfaction; increased staff confidence to perform healthcare roles; improved standard of healthcare delivery; and increased adoption of eHealth innovations beyond the health sector. Conversely, an outcome that was reversed following the discontinuation of SatCom from health facilities is the availability of accurate and reliable data for decision-making.

**Conclusion:**

Digital technology can have lasting impacts on health workers, patients, and the health system, through improving data management for decision-making, the standard of maternity service delivery, boosting attendance at health facilities, and utilization of services. Locally driven investment is essential for ensuring the long-term survival of eHealth projects to achieve sustainable development goals (SDGs) in LMICs.

## Introduction

The Alma-Ata Declaration of 1978 was a major milestone in the field of public health when countries of the world united to ensure health for all. It identified primary health care as the key to the attainment of the goal of Health for All. Ensuring healthy lives and promoting wellbeing for all ages is also a core statement of Sustainable Development Goals 3 (SDGs) (1–3). However, four decades after the Alma-Ata declaration and 6 years into the SDGs, many low- and middle-income countries (LMICs) of the world are still far from creating universal access and achieving the “health for all” goal for their citizens (4, 5). With a maternal mortality ratio (MMR) of 512 per 100,000 live births and an infant mortality rate of 67 per 1,000 live births (6), Nigeria has one of the worst maternal health indices in the world (7–9). A weak healthcare system, poor infrastructure as well as varied social and economic factors are challenges to attaining optimal healthcare system performance and improving maternal healthcare in the country (10). Also, inequity in the distribution of healthcare infrastructure in many sub-Saharan African countries has also widened the gap in realizing access to healthcare for most marginalized groups. The national healthcare structure is pyramidal, weakened at the village level, and disproportionately favors provincial and national hospitals (10). An alternative international standard for health professional education, particularly for local students from underserviced areas, which leverages the expertise of communities to identify community-specific health priorities has been recommended (11). While these problems persist, adopting e-health solutions has strong promises for scaling up healthcare services and improving the quality of services particularly to underserved populations (12).

E-health, defined by the World Health Organization (WHO) as the use of information and communication technologies (ICTs) for health (13) has enabled improved public health and primary healthcare services. E-health has been used for disease surveillance, primary health data acquisition and analysis, support of community health workers, teleconsultation, tele-education, research, and patient management (13). Globally, e-health is providing answers to many development questions. Some recent studies which focus on the impact of e-health have shown how technology-mediated strategies have helped to engage patients in modifying unhealthy behavior and improving medication adherence to reduce morbidity and mortality from cardiovascular disease (14, 15). Other studies have also shown that e-health has great promises at improving the skills, knowledge, and confidence of health workers and also enabling them to initiate and complete clinical tasks (16).

This study was part of a broader project that involved introducing computer-tablet-based video training and data digitization applications to healthcare facilities across three Nigerian states: Kano and Ondo state and the Federal Capital Territory over 2-years (March 2017–2019). The study protocol (12) and overall findings from the broader project (contextual enablers and impacts of using digital technology to extend Mother and Child Health (MCH) services to rural areas of Nigeria) (17) have been reported elsewhere. This study, therefore, focuses on assessing the sustainability of the impacts of introducing digital health innovations in 62 intervention healthcare facilities located in three local government areas (LGAs) of Ondo state supported by satellite communication (SatComs) technology combined with the existing 3G mobile network. Specifically, the aim of this study is to evaluate whether the expected project outcomes that were achieved at the end-line evaluation of 2019 were sustained 12 months after the project ended. The purpose of the study, sustainability of the impacts of digital health technology, is defined as the longevity and the continuing manifestation of the benefits and outcomes of digital innovations on health workers, the standard of healthcare, and patient experience long after the project closedown.

## Materials and Methods

### Study Setting

This study is set in Ondo State, western Nigeria. Ondo State has a population of 5.3 million and is made up of 18 local government areas (LGAs) with Akure as its capital city (18). The state has about 800 primary health facilities, 18 general hospitals, and 6 tertiary health facilities (19). The primary health facilities are managed by LGAs but are funded through statutory allocations from the Ondo state government. The doctor-patient ratio in the state is 0.71:10,000 population compared to 1:5,000 as recommended by the WHO. A recent report shows that human resources for health in the state include 148 medical doctors, 908 nurses, 137 medical laboratory technologists and scientists, 185 Community Health Officers (CHOs), and 1,152 community health extension workers (CHEWs) (19). There is an inequitable distribution of qualified health workers, especially doctors and nurses with higher proportions location in the urban compared to rural areas of the state. Regarding health indicators, Ondo State has a maternal mortality rate of 170/100,000 live births and an infant mortality rate of 49/1,000 live births in 2018 (6) compared with a national average of 512/100,000 and 67/1,000 live births, respectively. While the eHealth project's goal was to use digital technology to extend healthcare services to remote areas of Nigeria, Ondo state contained both rural and urban centers with a 70% rural and 30% urban divide. Similarly, the population of the state is split between those who are Christian (65%) and Muslim (35%) (1, 20). Ondo State was selected as the setting for investigating the sustainability of the impacts of eHealth as it had over 6 years of experience of adoption and implementation of digital technology in the health sector and the non-health sectors (e.g., for revenue generation) compared to Kano state and the Federal Capital Territory that had only 2 years experience of partnering with InStrat to adopt and implement digital interventions in the health sector (for maternal health services).

### Study Design

**Figure 1:**
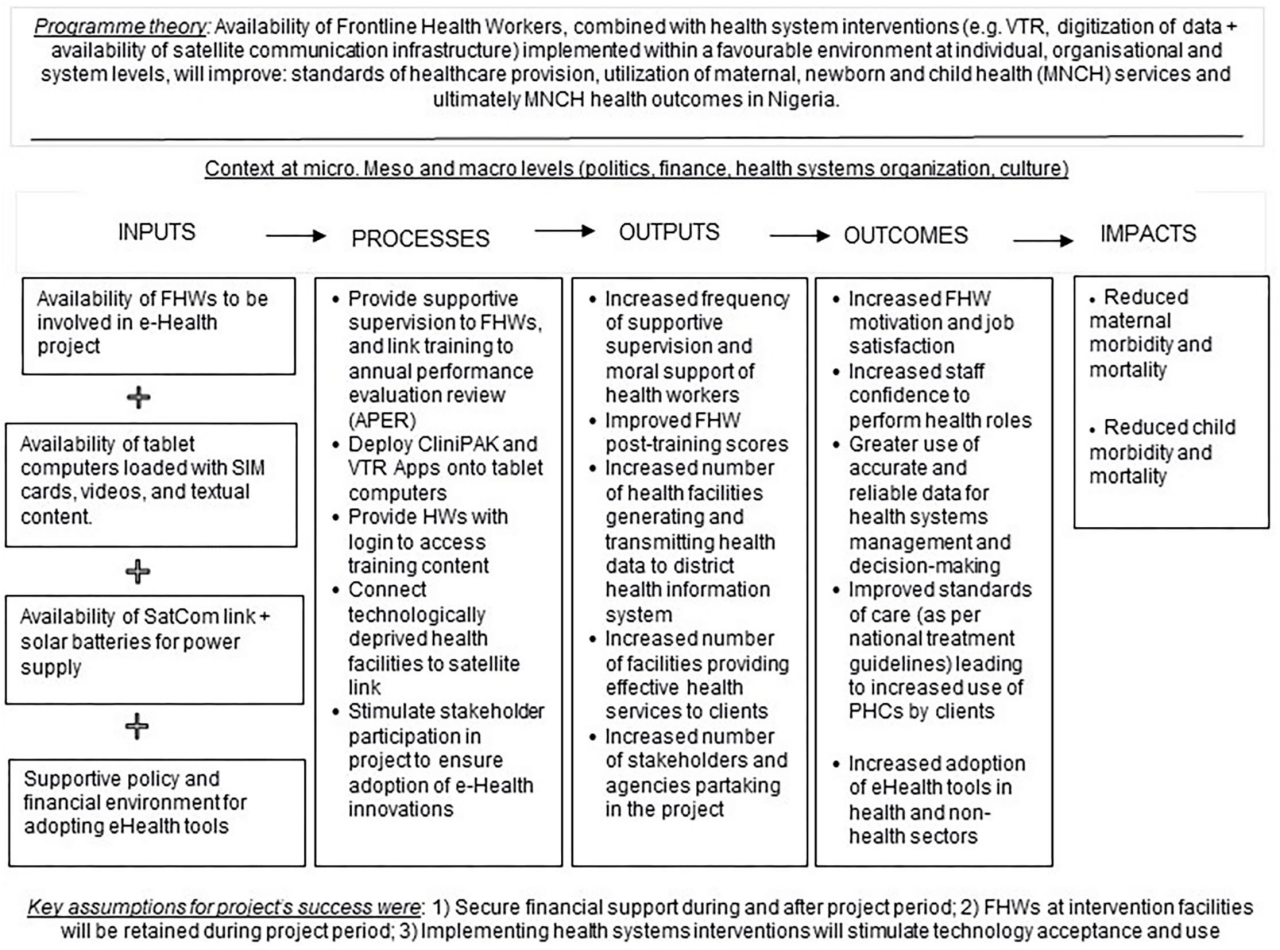
Theory of change (ToC) model for eHealth project in Nigeria.

A mixed-method design was adopted to evaluate the sustainability of the effects and impacts of implementing novel eHealth tools on MCH service delivery in Ondo state. Data collection for the legacy evaluation conducted 12-months post-project closedown combined (i) documents review combined with (ii) quantitative data extracted from health facility registers of patient care, and (iii) the collection of 24 most significant change (MSC) stories from stakeholders to assess the longevity of the impacts of eHealth tools on the standard of MCH care and other expected outcomes and impacts outlined in the project's Theory of Change (ToC; see Figure 1). For this reason, the legacy evaluation aimed to assess the extent to which project outcomes that were achieved at end-line evaluation (in 2019) were sustained beyond the project's lifetime. As part of the study design, we selected health facilities from different LGAs in Ondo State to facilitate the examination of different contextual factors that affected the implementation and project outcomes. The 2-year eHealth project (March 2017–2019) involved incrementally supplying 62 intervention Primary Health Care (PHC) facilities in 3 intervention LGAs across Ondo state with tablet computers loaded with data plans to enable the *eLearning* and healthcare data digitization interventions. Health workers in the 62 intervention facilities were then trained by InStrat (a local technology company that implemented the eHealth solutions in Nigeria) staff to use the tablets. Thirty-seven of 62 health facilities (59.7%) lacked access to a 3G mobile network and were supplied with a broadband global area network link-based SatCom hardware to facilitate internet connectivity in remote rural facilities. The remaining 25 facilities (40.3%) were connected *via* the regular 3G mobile network and so did not require linking *via* SatCom. See Table 1 for the distribution of health facilities and modes of delivery of digital technology in Ondo State. Intervention LGAs (Akoko south, Idanre, and Odigbo) were purposively selected for having the highest numbers of health facilities that lacked connectivity to a 3G mobile network in the state, while non-intervention LGAs (Akoko Northwest, Irele, and Ondo East) were randomly selected but shared similarity in the lack of accessibility to a 3G mobile network.

**Table 1:** Distribution of facilities and modes of delivery of eHealth tools by local government areas (LGAs) in Ondo State.

**Intervention LGAs**	**No of intervention health facilities**	**Modes of delivery of digital health tools and No of facilities using each mode**	**Non-intervention LGAs**	**No of non-intervention health facilities**
Akoko South	21	SatCom =11 3G mobile Network = 10	Akoko Northwest	25
Idanre	21	SatCom = 14 3G mobile Network = 7	Irele	21
Odigbo	20	SatCom = 12 3G mobile Network = 8	Ondo East	24
Total intervention LGAs = 3	Total intervention facilities = 62	Total SatCom = 37 total 3G mobile network = 25	Total non-intervention LGAs = 3	Total non-intervention facilities = 70

### Conceptual Framework

A ToC model for the project was co-designed with health workers and policymakers (12) to investigate how inputs and processes of the project led to outputs and how outputs led to outcomes and impacts (21). The ToC (in Figure 1) also outlines a program theory (see the upper part of Figure 1) of how the project was expected to generate a change along with contextual factors that can influence change.

Three central assumptions for the project's success that was extracted from document reviews and informal discussions with project stakeholders in 2017 were:

- Financial support will be secure for the project's 2-year duration (2017–19) and will be extended for another two years thereafter (up to 2021).
- Health workers in intervention facilities will not be redeployed during the project period.
- Implementing health systems interventions will stimulate technology acceptance and use.

Based on the significance of context to achieving project outcomes, we evaluated the role of SatCom, VTR, and data digitization interventions in achieving project outcomes within the implementation context (politics, national financial situation, culture, etc.) instead of attributing changes in outcomes to our project alone. The extent to which the central program theory outlined in the ToC (Figure 1) was borne out at the end of the project implementation was assessed during the end-line evaluation conducted in January 2019. The central proposition of the project was that: “Availability of Frontline Health Workers, combined with health system interventions (SatCom, VTR, and digitization of data infrastructure) deployed within a favorable environment at individual, institutional, and system levels will improve standards of MCH provision, utilization of health services, and health outcomes in Nigeria.” Five project outcomes and two impacts were outlined in the lower part of Figure 1 and assessed by the End-line evaluation. The project outcomes were: (i) staff motivation and satisfaction, (ii) increased staff confidence to perform healthcare roles, (iii) improved standards of care leading to increased attendance at PHC facilities and utilization of MCH services, (iv) greater use of accurate and reliable data for health systems management and decision-making, (v) increased adoption of eHealth innovations in health and non-health sectors. The impacts were: (i) reduced maternal morbidity and mortality and (ii) reduced child morbidity and mortality. Findings of the end-line evaluation conducted in Ondo state showed that all five outcomes above were achieved at the end of the project implementation in 2019. This study will therefore focus on assessing the extent to which the outcomes achieved during end-line evaluation in Ondo State were sustained 12 months after the project closedown.

To complement the above ToC model, we employed a modified technology acceptance model (mTAM) to explain the stakeholders' adoption of digital health interventions in Ondo states (22). The classic technology acceptance model (TAM) (23) outlines two main factors that shape people's intention to use technology. The first is the perceived usefulness of technology or the degree to which technology improves job performance. The second is perceived ease of use or the degree to which using technology is effortless. The ease of use of technology is thought to increase people's motivation to use the technology. However, in addition to classic TAM categories, the mTAM proposes that contextual factors within the setting that technology is implemented can influence people's perception of usefulness and adoption of technology (24). Findings of acceptance of VTR and data digitization interventions in Ondo state have been reported elsewhere as part of a broader project (17) and are therefore not repeated in this study.

### Sampling and Data Collection Methods

We used a combination of documents review, quantitative data extracted from facility-level registers of patient care, and collection of MSC stories from 4 service users, 4 community members, 6 Frontline Health Workers (FHWs), 6 facility managers, and 4 policymakers to assess the sustainability of the impacts of digital health interventions. Purposive sampling was used to ensure that all five groups (service users, community members, health staff, facility managers, and policymakers) were represented in the interviews. All participants were at least 18 years of age. See Table 2 for details of stakeholders recruited for data collection. MSC stories were conducted by a male sociologist (DA) and two male medical doctors (KO, AA), trained in qualitative interviewing. Data collectors also attended three sessions of online training in the first week of February 2020 on the use of the MSC methodology for the legacy evaluation. The sessions of training were facilitated by BE, who is experienced in using the MSC methodology. Qualitative data were collected from 4 health facilities (2 Primary Healthcare Centres and 2 Comprehensive Health Centres) from the 4 LGAs selected for the legacy evaluation.

**Table 2:** Stakeholder groups interviewed for legacy evaluation.

**Stakeholder group**	**Intervention *LGAs***	**Control *LGAs***	**Total (%)**
Service users	2	2	4 (16.6)
Community members	2	2	4 (16.6)
Health workers	4	2	6 (25%)
Facility heads	4	2	6 (25%)
Policy makers	2	2	4 (16,6%)
Total	14 (58%)	10 (42%)	24 (100%)

Data collectors provided potential participants with the study information sheet to explain study objectives and help participants decide to participate (or not) in the study. Participants were given at least 24 h to decide to partake in the study. The guides for collecting stories of MSC were pre-tested before they were administered on the field. See Supplementary Material 1 for the example of guides for collecting stories of MSC. The interviews lasted from 20 to 30 min and were conducted in a private setting in a health facility, audio-recorded, transcribed verbatim, and where appropriate translated into English for analysis. As the eHealth project aimed to use digital technology to improve MCH services in rural areas, the documents reviewed as part of data collection for the legacy evaluation included: (i) facility-level registers to verify healthcare services rendered, (ii) state MCH policies, (iii) ICT policies, and (iv) reports of MCH programs to understand how the eHealth project was meant to work and the assumptions held by stakeholders. The documents reviewed by the authors of this paper were used both as evidence for study findings (e.g., quantitative data extracted from facility-level registers) and as an explanation for the study findings (e.g., data from eHealth policies).

### Stakeholders Definitions

The five groups of stakeholders who were approached and recruited were: (i) service users (pregnant women and new mothers) who used health facilities within the last 12 months of each evaluation, (ii) FHWs, and (iii) facility managers who worked within health facilities in both intervention and control arms of the study, (iv) community members who were representatives of ward health committees that participated in implementing healthcare activities and were tasked to interface with the primary health care facility, and (v) policymakers who were responsible for making and implementing maternal and reproductive health policy in the state.

### E-Health Interventions

The digital health interventions involved providing 62 facilities with a tablet computer containing a video training application (VTR Mobile) and a data digitization application.

**a) VTR Mobile Application**: allowed participants to access video, audio, and text-based MCH materials through the internet. The videos used were developed by *Medical Aid Films* (https://www.medicalaidfilms.org) and the *Global Health Media Project* (25) and accessed through the *ORB Platform* (www.health-orb.org/), but developed jointly with the *mPowering Frontline Health Workers Partnership* (26). The ORB platform hosts high-quality medical content that can be used under a creative commons license to train frontline workers *via* the internet or *via* downloads to mobile devices (12). The videos were delivered to users *via* a structured VTR Mobile program and provided clear educational content and captivating MCH-related clinical scenarios including antenatal care, care during labor, and postnatal care. Video contents used for the study were selected jointly with the officials of the Ondo State Ministry of Health.

**b) Data Digitization Application:** was a computer-enabled decision support and point-of-care data capture solution that enabled FHWs to capture patient-level health information and transmit relevant healthcare data to remote servers *via* mobile networks. The digitization software also served as an electronic medical record (EMR) for gathering data on patient demography, vital signs, clinical diagnosis, the treatment recommended, and administrative task support. The software triggers alerts for at-risk patients for possible referrals to secondary health systems and on-demand reporting to support administrators to increase productivity and improve patient clinical experience. The digitization software and content are developed and owned by Vecna Cares Charitable Trust, Massachusetts, USA, but the app uses locally available technologies and infrastructure to transmit medical records to remote computer servers while making data available for decision support and project management.

The staff of the e-Health interventions provider (InStrat Global Health Solutions) provided login details to study participants to facilitate login and use of VTR and data digitization applications, which also tracked usage of the apps.

### Most Significant Change Methodology

The MSC technique is a form of participatory monitoring and evaluation approach that involves project stakeholders in deciding the sorts of change to be recorded and in analyzing the data (27). The legacy evaluation exercise adopted the MSC method for qualitative data collection which was a slightly different method from the traditional qualitative interview method used during the end-line evaluation conducted in March 2019. Unlike traditional qualitative interviews, the MSC approach is based on storytelling which should lead to the critical question “what changes occurred?,” in terms of which actors did what, when, how, and why are their actions important? (2, 28). The MSC approach collects personal stories that reflect on individual experiences and/or observations of change over time (29–31). We used the MSC methodology (29) to identify and understand changes that study participants and stakeholder groups attributed to the digital interventions in the 12 months following the withdrawal of funding from the eHealth project. Three data collectors (AA, DA, and KO) interviewed FHWs, facility heads, service users, community members, and policymakers asking them to tell us stories of the most significant change(s) they had experienced and/or observed as a result of deploying video training and the digitized application for data management. Supplementary Material 1 provides details of the questions and prompts asked of the stakeholder groups. The MSC approach involved a series of steps including: (1) raising the interest of stakeholder groups about the use of the MSC method for the legacy evaluation; (2) defining the domains of change that stakeholder groups are interested in; (3) defining the time frame for analyzing stories; (4) collecting “significant change” stories from stakeholder groups; (5) discussing all stories and voting for the most significant stories and why they were selected; (6) and providing feedback on the results of the selection process to participants to facilitate inclusion of important contextual details.

We adopted a participatory process to define domains of change with stakeholder groups being asked to suggest domains of change and what constituted the change that they thought was significant (32). This process led to the identification of four board domains of healthcare used to assess the longevity of outcomes of digital health innovations: (i) standard of maternity care, (ii) staff attitude and behavior toward patients, (iii) use of eHealth technology for data management, and (iv) utilization of health facilities by patients. The first two domains were suggested by service users and community members while the latter two domains were suggested by health workers and policymakers. It is important to highlight that “domains of change” *are not* pre-determined indicators of success selected in a top-down fashion by project implementers and/or evaluators for monitoring and evaluating projects. By contrast, domains of change are usually defined so that everyone can interpret them in the same way, while being fuzzy to enable stakeholder groups to hold different interpretations of what constituted the change that they thought was important.

### Data Analysis

**For quantitative data:** We analyzed quantitative data extracted from facility-level registers using an Excel spread sheet to present results in graphical formats. Data extracted from health facility registers were compared for concurrence with Ondo state Health Management Information System data. **For qualitative data:** Stories of most significant changes collected as part of the legacy evaluation were audio-recorded, transcribed, and where data collection was conducted in Yoruba (the local language), they were translated into English for analysis. Manual contents analysis was used to organize data from the MSC stories into themes to enable stakeholders to rank and vote for excerpts with the most significant change stories of the interventions. The ranking and voting process was originally conceived to be conducted at four levels: by health workers, policymakers, the University of Lagos research team in Nigeria (AA, DA, and KO), and by the University of Leeds research team in the UK (BE and MJA). However, this process was later condensed to two ranking and voting levels only due to the outbreak of COVID-19 during the fieldwork in March 2020. The criteria for voting were organized around the themes of *relevance* and *impact* elicited from the contents of stories collected from stakeholders. It is important to emphasize that the pandemic did not affect data collection for the legacy evaluation, as data collection was completed on March 19, 2020, while national lockdown restrictions were enforced on March 30, 2020. Based on identified themes and criteria for ranking, stories were sent to researchers who ranked in order of significance by voting from 1 to 3; with 3 being the most significant change story, and 1 being the least significant change story. AA, DA, and KO of the University of Lagos Research Team participated in the voting exercise, following their initial ranking of each story from 1-3. For each theme, the ranked scores were aggregated and stories with the highest score were selected as the most significant. Thus, the theme with the largest number of stories with significant change was voted the most impactful theme. AA, DA, and KO were supported in the task of ranking and voting for stories through attending the third of three online sessions. This third session was dedicated to selecting and voting for MSC stories. The reading and reference materials circulated ahead of the training sessions included a Practical Guide for the MSC Technique, a Framework for writing stories of change, and a Guide for story selection and ranking processes. Following their training, AA, DA, and KO ranked and voted for MSC stories over two Zoom video calls (the first call for selecting and ranking stories, while the second call for voting for most significant stories). The two Zoom calls facilitated discussions and interaction among team members about reasons for selecting and ranking stories.

Multiple datasets including quantitative data extracted from facility-level registers and qualitative data extracted from transcripts of stories of the most significant changes were repeatedly analyzed and triangulated to facilitate a better understanding of the sustainability of the impacts of digital health tools on health worker knowledge, the standard of health service provision, and client experiences. Manual data analyses of MSC stories were led by AA, DA, and KO who interviewed stakeholder groups (service users, community members, FHWs, facility managers, and policymakers). Analyses were then verified by BO, BE, and MJA and approved by all six authors to make sense of the sustainability of impacts of eHealth interventions and contextual factors that influenced project results. All authors read and re-read transcripts for MSC stories to be thoroughly immersed in the data. Five transcripts (representing the five stakeholder groups) were then randomly selected and used to develop a coding frame by DA and BE, who met *via Zoom* to compare their findings and to decide which categories to use for coding the remaining data from stakeholder transcripts. Questions used to interrogate the five transcripts and which fed into the identification of categories were: Why are selected stories significant? What differences are there between stories selected from different health facilities? What differences are there between MSC stories told by different stakeholder groups? This process was then repeated till all remaining 19 transcripts were coded, necessitating expansion of the coding frame with new categories in the data. The findings reported below describe stakeholder responses using themes and subthemes identified and align with the consolidated criteria for reporting qualitative research—COREQ—see Supplementary Material 3 (3, 33).

## Ethics Approval

Approval for the study was granted by the University of Leeds School of Medicine Research Ethics Committee (MREC16-178) and the research ethics committee of the Ondo State Ministry of Health (AD.4693 10 Vol. II/109).

## Results

### Socio-Demographics

A total of 24 stakeholders were interviewed during the legacy evaluation activities. The gender composition of respondents shows that more females (*n* = 16; 66.7%) participated in the study. This was expected as the majority of service users and personnel in health facilities (FHWs and facility heads) were females. The majority of respondents were aged between 30 and 49 years (*n* = 15; 62.5%). See Table 3 for details. More health workers (*n* = 6; 25%) and facility heads (*n* = 6; 25%) participated in the study than community members (*n* = 4; 16.6%), service users (*n* = 4; 16.6%) and policymakers (*n* = 4; 16.6%).

**Table 3:** Characteristics of stakeholders interviewed for legacy evaluations.

**Socio-demographic attributes**	**Frequency**	**Percentage**
**Gender**		
Male	8	33.3
Female	16	66.7
**Total**	**24**	**100.0**
**Age**		
25–29	4	16.7
30–34	2	8.3
35–39	3	12.5
40–44	4	16.7
45–49	6	25.0
50+	5	20.8
**Total**	**24**	**100.0**
**Designation**		
Service user	4	16.7
Community members	4	16.7
Policy maker	4	16.7
Health worker	6	25.0
Facility head	6	25.0
**Total**	**24**	**100.0**
**Education**		
Primary	3	12.5
Secondary	5	20.8
Tertiary	16	66.7
**Total**	**24**	**100.0**
**Marital status**		
Single	2	8.3
Married	20	83.4
Widowed	2	8.3
**Total**	**24**	**100.0**

Majority of respondents had tertiary (*n* = 16; 66.7%) and secondary education (*n* = 5; 20.8%), respectively. A large percentage of respondents were married (*n* = 20; 83.3%). The majority of stakeholders had positive perceptions of digital health technology and affirmed the relevance of the Satcom, VTR, and data digitization interventions for enhancing healthcare delivery in Ondo state, especially in rural areas. The aspects in which the project led to significant changes and how it impacted the healthcare delivery in the state are discussed next. The sections that follow outline project outcomes of deploying digital health interventions (SatCom, VTR, and data digitization technologies) which were achieved during the end-line evaluation conducted in January 2019 and outlines the extent to which the achieved outcomes were sustained after 12 months of project closedown.

### Expected Outcomes and Impacts Achieved at End-Line Evaluation

As highlighted already, the findings of the End-line evaluation revealed all five project outcomes outlined in the ToC model (Figure 1) were achieved in 2019 namely: (i) staff motivation and satisfaction, (ii) increased staff confidence to perform healthcare roles, (iii) improved standards of care leading to increased attendance at PHC facilities and utilization of MCH services, (iv) greater use of accurate and reliable data for decision-making, and (v) increased adoption of eHealth in health and non-health sector. In subsequent paragraphs, we will draw on findings from the legacy evaluation to highlight the extent to which the outcomes that were met during the end-line evaluation were sustained beyond the lifetime of the project.

### Main Themes From the Legacy Evaluation

The results are further categorized into (i) outcomes that were sustained 12 months after funding was withdrawn from the eHealth project in March 2019 and (ii) outcomes that were reversed. The outcomes that were sustained include staff motivation and satisfaction; increased staff confidence to perform healthcare roles; improved standard of care; and increased adoption of eHealth in health and non-health sectors. Conversely, the outcome that was reversed is the availability of accurate and reliable data for decision-making. These are discussed next using quotations and/or facility-level quantitative data as supporting evidence where necessary.

#### Sustained Outcomes

##### a) Staff Motivation and Satisfaction

Qualitative analysis found that prior to the introduction of digital technology in 2017, there was a gross shortage of skilled FHWs, as well as weak and irregular in-service training of health workers, especially in the hard-to-reach areas of Ondo State. This underscored the relevance of the VTR component of the project as a disruptive innovation. Akin to the findings of the end-line evaluation, stories of MSC collected during the legacy evaluation revealed that access to clinical videos improved knowledge and skills of FHWs, which in turn manifested as staff motivation and satisfaction. A key impact of VTR cited in the stories of MSC was that VTR promoted the decentralization and democratization of training by enabling FHWs to access high-quality training content on tablet computers and reduced the need to travel to capital cities to attend training. In this sense, VTR helped to bridge the gap in skilled manpower especially in the hard-to-reach areas of Ondo state.

##### b) Increased Confidence to Perform Healthcare Roles

In addition to enhancing knowledge and skills, 4 of the 6 health workers interviewed reported that accessibility to VTR led to increased staff confidence to handle medical cases they were previously afraid to handle as seen in the quote below:

Before this project, whenever I came to work and I heard that a woman was in labor, I was scared (because I didn't know what to do). But since I started watching the VTR videos, I can take delivery without issues (i.e., much fear). I love my job more now because watching these videos have increased my knowledge and boldness to interact with patients and to take deliveries (*Health worker, Onikokodiya_health facility Idanre LGA)*.

##### C) Sustained Improvement in the Standard of Care

The analysis of stories of MSCs during the legacy evaluation identified that the high-quality service delivery reported during end-line assessment was sustained 12 months after the project ended. A key finding of the legacy evaluation was that watching clinical videos empowered many low cadre health workers (CHEWs) with skills and competence to provide maternity services including using partographs to monitor and track the vital signs of women in labor. In describing the successes achieved in safely delivering pregnant women in her health facility following the introduction of partographs in 2017, a facility head said:

Before this project, we didn't use partographs for taking delivery here, but after watching the videos on VTR, the videos taught us how to use partograph, how to chart the patient data, including guiding us to know the time we are supposed to check the patient, the time we are supposed to report what we find in the chart. If the patient is not up to 4 cm dilated, we should not touch the partograph. After learning all that, I showed all my colleagues how to use partograph to be taking delivery *(Facility Head in Aseigbo LGA)*.

**Figure 2:**
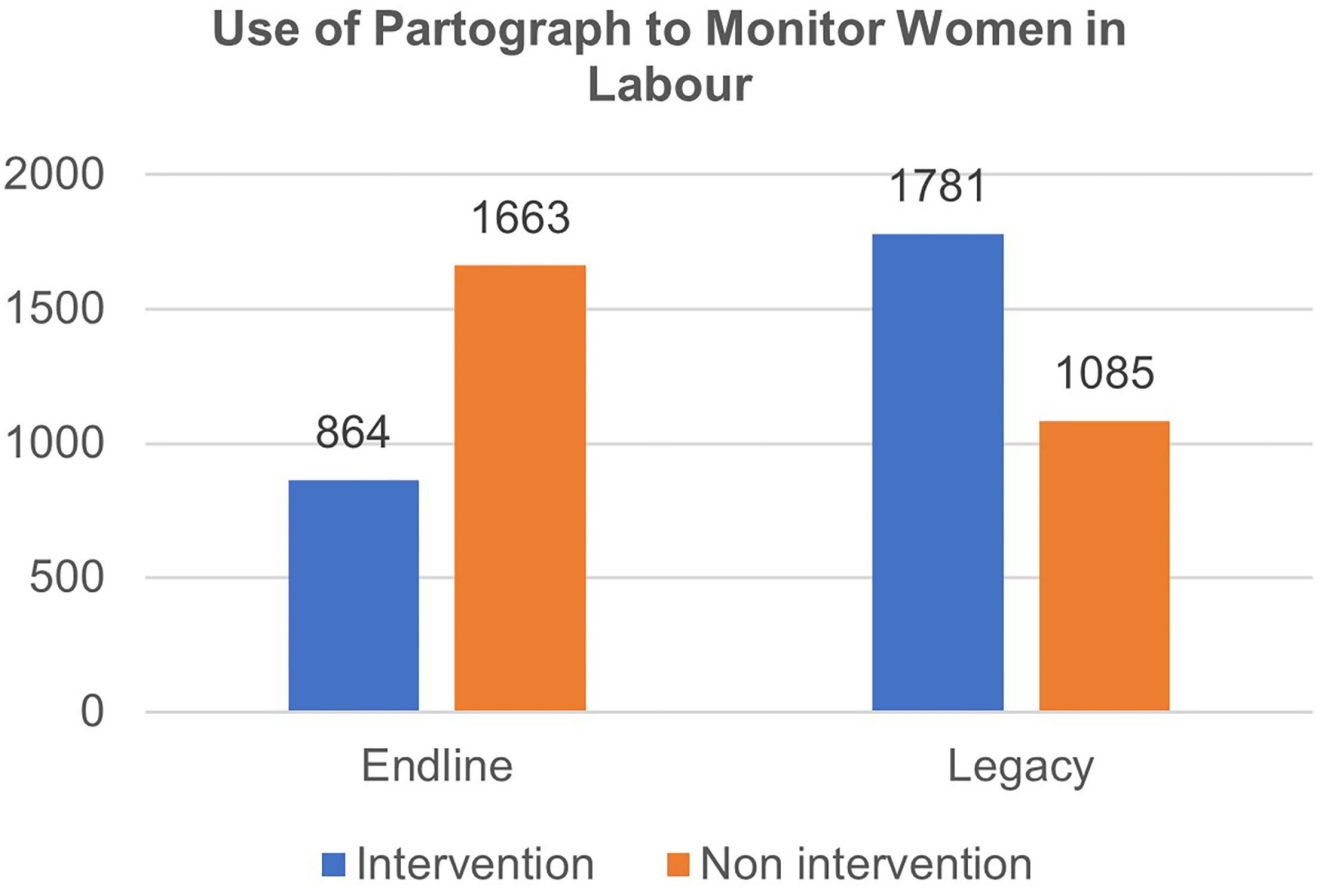
The use of partogram to monitor labor at end-line and legacy evaluation.

To corroborate the above narrative, quantitative data extracted from facility-level registers for managing women in labor showed that the use of partograph for monitoring delivery at intervention sites more than doubled between the end-line evaluation in January 2019 and the legacy evaluation exercise in March 2020 (see blue bars in Figure 2).

In specific terms, the number of births monitored using partographs increased by 106% at intervention sites, from 864 deliveries monitored in 2019 to 1781 deliveries in 2020; whereas non-intervention sites recorded a 34.8% reduction in numbers of deliveries monitored with partographs from 1,663 in 2019 to 1,085 deliveries in 2019.

A facility head recounted how VTR impacted the FHWs competence and performance:

In the past, when we took deliveries, we didn't usually clean the baby like that (as in the video). So, we used to just cut the cord, then oil the baby, pack the baby and clothe the baby. But when we watched VTR, we saw that when you deliver a baby, you should take the baby to the mother first, then you clean with dry cloth, then you wrap the baby. You never just cut the baby's cord immediately. Then after that, that baby will stimulate the breast, where the breast is and it can begin to suck the breast. Then after that, when you cut the cord you now take the breast to the child. That is one particular thing that I've learned from that video that has improved my ability to save the lives of babies *(Facility Head in Idanre LGA)*.

This shows that significant gains and progress in MCH are achievable with simple innovations aimed at enhancing the standard of care and upgrading the skills of frontline health workers. A related significant change attributable to the eHealth project was the use of clinical videos for health promotion in antenatal care (ANC) clinics, which in turn increased patient awareness of the importance of ANC and indirectly increased attendance at ANC clinics.

**Figure 3:**
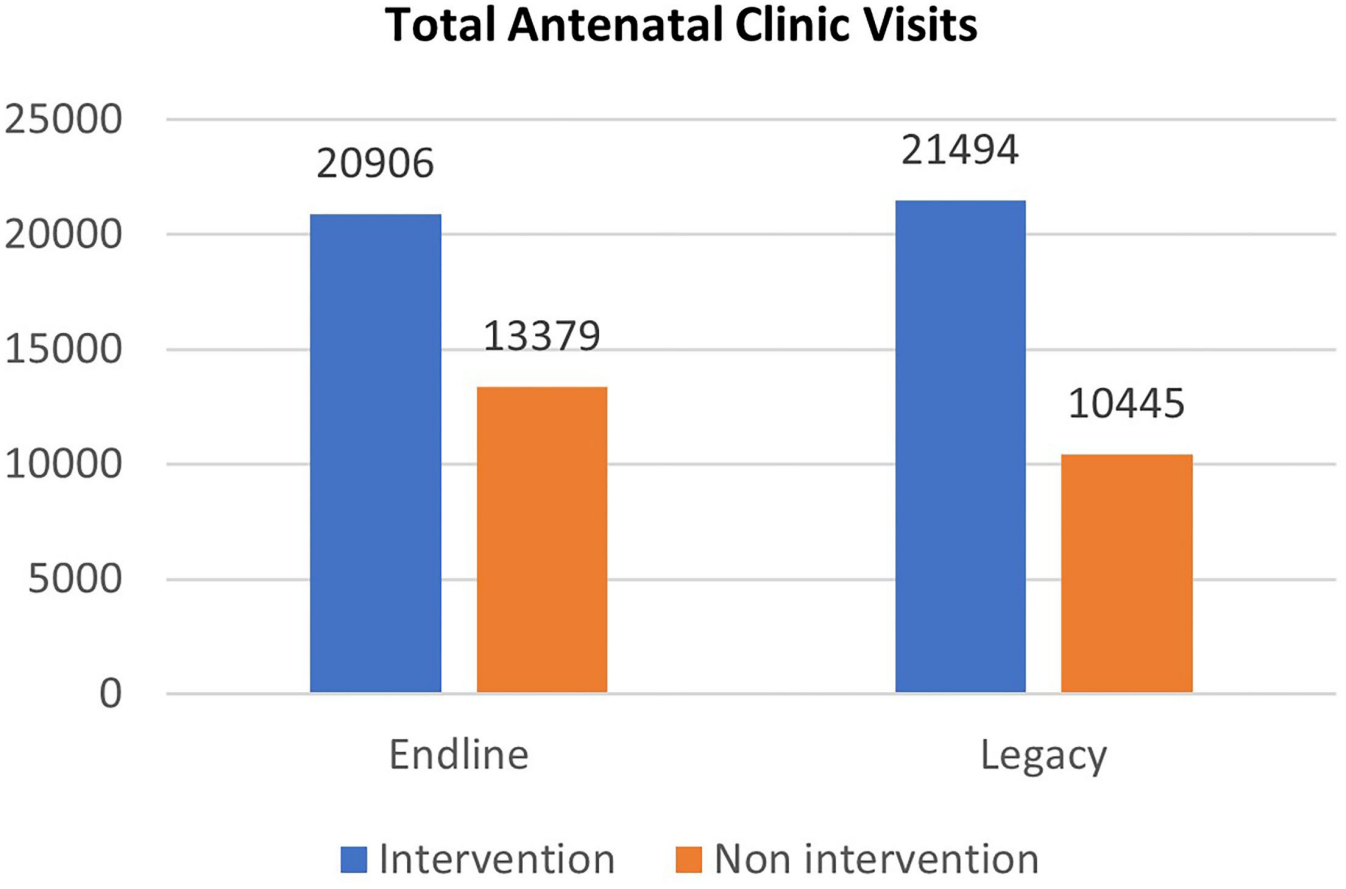
Antenatal clinic visit across two phases of evaluation.

Figure 3 shows that the numbers of pregnant women attending ANC clinics at the intervention sites increased by 2.8% from 20,906 attendances at end-line to 21,494 attendances (at legacy), whereas there was a 21.9% reduction in ANC attendance in the non-intervention facilities to 10,445 visits (at legacy evaluation) from 13,379 visits at end-line. The analysis of the stories of MSC suggests that pregnant women who watched clinical videos at ANC clinics reported improved understanding of personal hygiene, appropriate diet, and self-care during pregnancy that inspired them into action. A service user reported how watching some clips on the VTR had changed her perspective on basic hygiene practices during pregnancy and helped her to improve her diet during pregnancy.

You know when we are in the village, we act like village folk, but after watching this video, we have seen how we can change, like how we can take care of our homes even if we are living in a one-room house. If we used to have a dirty manner, the video has shown us what to do to change such practices because of our health and health of the baby *(Service user in Ondo state)*.

It was common practice for those who watched the videos to go back to the community to sensitize other women who, in turn, visited health centers to learn more about pregnancy care. This unexpected but innovative use of VTR in ANC clinics led to a remarkable increase in ANC attendance in health facilities in Ondo state. One health worker shared her experience on how the VTR facilitated the mobilization of pregnant women to attend health facilities during pregnancy:

One thing that has happened since the project started is that it (VTR) helped us to mobilize our community, and the patients are patronizing us. If any woman gets pregnant and did not want to register here, some of the pregnant women that have been coming here will tell their neighbors…like… “when you go for that clinic, look out for the video there,” so that has rallied pregnant women in our community even those mothers, who did not register here before *(Health worker in Odigbo LGA)*.

##### d) Increased Adoption of eHealth Interventions in Ondo State

Insight from the stories of MSC suggests that implementing multiple digital health interventions for 2 years within a supportive policy environment in Ondo State had generated significant interest in government and from other stakeholders in the health sector to adopt eHealth innovations. An example of increased adoption of eHealth innovations by the Ondo State Government is the launch in December 2019, of a new ***electronic Contributory Health Insurance Scheme (eCHIS)*** as a pathway to promoting equitable access to essential healthcare services and accelerating universal health coverage (34).

#### Reversed Outcome: Use of Accurate and Reliable Data for Decision-Making

Apart from positive outcomes, findings from the legacy evaluation also identified an outcome that was reversed within 12 months of the project's closedown. However, unlike findings of the end-line evaluation that reported the viability of SatCom technology for providing uninterrupted connectivity to health facilities in technologically disadvantaged areas of Ondo state, interviews with stakeholders during the legacy evaluation found that the SatCom technology had, in August 2019, been disabled in all 37 health facilities (see Table 1) following the closedown of eHealth project. FHWs interviewed in March 2020 lamented how the discontinuation of SatCom intervention had significantly affected the capacity of health facilities to generate, manage, and transmit timely and reliable data to LGA, state, and national levels to support decision-making. An FHW's comment is captured below:

Everything we do used to be by pressing that tab on the computer tablet, then the information will go directly to the dashboard and the people in the LGA will see our data. But this time now (since the project ended), the LGA officers complain that they don't see our information again in the dashboard. So, we have started sending hardcopy registers to the LGA headquarters for validation monthly. We send registers of all the activities that are carried out here to the headquarters. At times when we don't send our data to the headquarters in time, they will be calling me by phone saying: “we have not seen your work, please send your registers to us” (FHW in_Odigbo LGA).

Another FHW described how the discontinuation of Satcom caused their inability to use the data digitization App for data management in the state:

“The work has not been easy (since the project ended) because, you know, we will be looking for the case notes of patients one by one to be able to collate our data, whereas when we were using the data App, “you will just click on it, and you know, the data will come out, with the date you saw the patient and you will just click on it and it will display everything you are looking for”* (FHW in Aseigbo LGA)*.

To corroborate the above experiences about working without the data digitization app, a facility head described how the gains made during the eHealth project has been reversed within a few short months:

Before the project started, we used to carry registers (in huge bags) from this health center to go and do validation (at the LGA headquarters) and all the summary of the work is there…And during the eHealth project, we have been using it (data digitization app), and we noticed that our work was better and there is no stress for us.” (But after the project stopped) we cannot do our work well-again, we cannot send data with *I-pad* again. That is it!! …Although data is still being collected, it is no longer as comprehensive as before *(Facility Head in Aseigbo)*.

The last two quotes capture the frustration associated with reverting to manual data entry and of physically transporting hardcopy registers regularly to LGA headquarters for verification.

A major advantage of the data digitization application was that by acting as a decision-support tool, it increased efficiency and effectiveness by accelerating FHWs' ability to make diagnoses and prescriptions. In substantiating the app's impact on quality of care, one facility head said: “*we did not know what we were doing until the application was introduced.”* This view was supported by views from many health workers:

Ha! The App has really empowered us! Although the tablet computer is very small, but when you are operating it, it will guide you (on what to do). It will request for the name of patient, the address, and everything. Then in terms of diagnosis it will direct you, based on the complaint of the patient you will know the type of diagnosis that you give even to the drug available in the pharmacy. And even if you suppose to refer the patient you will let you know that you suppose to refer the patient *(Facility Head in Odigbo)*.

The foregoing benefits were unfortunately reversed within 12 months of discontinuing the data digitalization app, thereby forcing frontline health workers to revert to the inefficient old system of using hardcopy consultation folders and clinic registers for decision-making.

## Discussion

While global optimism for the potential value of digital health solutions inspired the growth of pilot eHealth projects, many pilot projects have not translated into long-term healthcare services. Such failures have generated skepticism among policymakers about investing in digital innovations. Similarly, while there is increasing interest in evaluating the effectiveness of digital health technology, there is limited guidance on evaluating the sustainability of the effects and benefits of such interventions. To fill this gap in the literature, this study aimed to evaluate whether the outcomes of deploying multiple digital technologies achieved at end-line evaluation in Ondo State Nigeria were sustained 12 months after the project ended in March 2019. This is one of few studies to report the evidence of sustainability (35, 36) of the effects of digital technology on service users, health workers, or the health system in LMICs.

As the majority of evaluations of the outcomes of digital health interventions are often conducted during the lifetime of digital health projects, the strength of this study is in using the MSCs methodology (as part of a mixed-methods design) to collect stories from five stakeholder groups to assess the longevity of project outcomes, revealing that four of the five outcomes that were achieved at end-line evaluation were sustained beyond the lifetime of the project. The sustained outcomes are: (i) staff motivation and satisfaction; (ii) increased staff confidence to perform healthcare roles acquired through continuous offline access to high-quality MCH-related training content streamed via the VTR app to health workers; (iii) improved standard of care which generated increased patient awareness of the benefits of attending ANC clinics following patients watching clinical videos during ANC classes which in turn led to positive behavior change and increased attendance and utilization of MCH services in Ondo state; (iv) and increased adoption of eHealth in health and non-health sector. An outcome that was reversed following the withdrawal of project funding was the availability of accurate and reliable data for decision-making. These findings have implications for policy and practice. First, it is not sufficient to only assess the effectiveness of digital health interventions during the period of implementation of the interventions. Second, the longevity/sustainability of expected project outcomes can be tracked beyond the lifetime of the project using approaches such as the MSC methodology as part of the repertoire of evaluation methods for the legacy evaluation of eHealth projects conducted at least 12 months after the project closedown. Third, significant positive improvements and progress in maternal and child health are achievable beyond the lifetime of eHealth innovations following continuous and simultaneous implementation of multiple context-specific digital innovations (16) aimed at enhancing standard healthcare services and upgrading the skills and performance of health workers.

Despite a supportive ICT policy environment in Ondo State coupled with verbal expressions of ownership of the eHealth project, there was a lack of financial investment in the project to guarantee its survival following the withdrawal of donor funds. This underpins the importance of locally driven long-term investment for the survival of eHealth technologies in LMICs (16). While global funding of health-related problems is shrinking (37), there is a renewed interest in developing sustainable funding models that leverage better management skills and efficient deployment of human and material resources which is highlighted by our study from Nigeria (17, 38). The lack of preparation of the government to assume financial responsibility for the eHealth project after donor funding elapsed in March 2019, demonstrates the centrality of embedding exit strategies into memoranda of understandings that specify project durations and who will be responsible for the project takeover after their completion. Furthermore, exit strategies should be discussed at project review meetings to adequately prepare governments to commit funding to the sustainability of projects.

This study from western Nigeria demonstrates that sustained and simultaneous implementation of multiple digital health technologies over 24 months have the potential: (i) to scale up knowledge, (ii) to mobilize and sensitize clients at the community level to utilize health services, (iii) to efficiently improve decision-making through diagnostic and therapeutic accuracy, and (iv) to improve the standard of care. While sustainability might be a temporal challenge, the benefits of multiple digital technologies reported by this paper can have long-term and transgenerational impacts for stakeholders. For health workers who benefitted from improved knowledge following continuous access to VTR over 2 years, there are huge potentials for long-term skills transfer to and motivation of low cadre staff. This suggests that sustained deployment of digital technology can enhance task sharing among multi-cadre health workers (e.g., from nurses and midwives to CHEWs), especially in settings with human resource constraints (39). Similarly, the benefits to pregnant women and new mothers who learned basic hygiene practices and safe food processing tips during health promotion classes can accelerate the achievement of UN Sustainable Development Goals. Notable for pregnant women and their families is goal 3 of ensuring healthy lives and improving wellbeing; and for female FHWs is goal 5 of achieving gender equality through empowering FHWs with knowledge, confidence, and clinical skills.

### Study Limitations

This study has three key limitations. First, the non-inclusion of pregnant women and service users in developing the ToC model may have led to the project overlooking the views and needs of patients and community members. Second, we acknowledge that purposive selection of three intervention LGAs with the highest numbers of health facilities that lacked access to a 3G mobile network potentially introduced bias in sampling. Nevertheless, the sampling decision was justified given the project goal of using SatCom technology to extend healthcare services to technologically disadvantaged facilities and areas of Nigeria. Third, while every effort has been made to ensure rigorous data analysis and triangulation of findings from multiple datasets (documents review, quantitative data from health facility registers, and reports of MSC stories), we acknowledge that the study is limited by the truncation of the voting process for MSCs stories from four levels to two levels prompted by the COVID-19 restrictions implemented in Nigeria from March 2020. The inclusion of health workers, policymakers, and community members in the voting, feedback, and verification of MSC stories would have further strengthened the findings in two ways: (i) help to minimize bias toward selecting/voting for popular views and excluding stories that harshly criticized health workers and policymakers; (ii) help to reduce subjectivity in story selection that can reflect the values of those on selection panels. However, the fact that we pre-recorded and agreed on the reasons for selecting stories and “domains of change” helped to strengthen the process and increased the objectivity of the process. Despite the above limitations, we have developed rich contextualized perspectives of the work evaluated.

## Conclusion

The findings of this study demonstrate the value of using the MSCs methodology as part of legacy evaluations conducted at least 12 months after the project closedown to understand and document evidence of the sustainability of outcomes and impacts of digital technology and to track progress toward the achievement of SDGs. Locally driven investment is essential for ensuring the long-term survival of eHealth projects to achieve SDGs in LMICs.

## Data Availability Statement

The original contributions presented in the study are included in the article/Supplementary Material, further inquiries can be directed to the corresponding authors.

## Ethics Statement

Ethical approval for the study was granted by the University of Leeds, School of Medicine Research Ethics Committee (MREC16-178), the Ondo State Government Ministry of Health (AD.4693 Vol. II/109). The patients/participants provided their written informed consent to participate in this study.

## Author Contributions

BE and BO jointly conceived the study. DA developed the manuscript. DA and BE led the writing of this paper with contributions from BO, AA, KO, and MA. All authors contributed to the article and approved the submitted version.

## Funding

This article presents independent research funded by the UK Space Agency International Partnership Programme, Grant reference number IPPC1-30. The sponsor, funders, and the company that implemented the technologies were not involved in the study design, collection, management, analysis, and interpretation of data, writing of the report, and the decision to submit the report for publication.

## Author Disclaimer

All views expressed in this publication are of the authors only.

## Conflict of Interest

The authors declare that the research was conducted in the absence of any commercial or financial relationships that could be construed as a potential conflict of interest.

## Publisher's Note

All claims expressed in this article are solely those of the authors and do not necessarily represent those of their affiliated organizations, or those of the publisher, the editors and the reviewers. Any product that may be evaluated in this article, or claim that may be made by its manufacturer, is not guaranteed or endorsed by the publisher.
