# Supplementary material for: Sustainability of the Effects and Impacts of Using Digital Technology to Extend Maternal Health Services to Rural and Hard-to-Reach Populations: Experience From Southwest Nigeria
Source: Front Glob Womens Health. 2022 Feb 8;3:696529. doi: 10.3389/fgwh.2022.696529 (PMC8861509; doi:10.3389/fgwh.2022.696529)
Supplement: Supplementary file 1 [file Data_Sheet_1.PDF]

Manuscript: Sustainability of the effects and impacts of using digital technology to extend maternal health services to rural and hard-to-reach populations: Experience from southwest Nigeria

Consolidated criteria for reporting qualitative studies (COREQ): 32-item checklist

Developed from:
Tong A, Sainsbury P, Craig J. Consolidated criteria for reporting qualitative research (COREQ): a 32-item checklist for interviews and focus groups. *International Journal for Quality in Health Care*. 2007. Volume 19, Number 6: pp. 349 – 357

No. Item	Guide questions/description	Reported on Page #
**Domain 1: Research team and reflexivity**		
Personal Characteristics		
1. Inter viewer/facilitator	Which author/s conducted the interview or focus group?	Pages 6 and 8 (section 2.4 data collection and section 2.8 data analysis)
2. Credentials	What were the researcher's credentials? E.g. PhD, MD	Page 6 (section 2.4 data collection)
3. Occupation	What was their occupation at the time of the study?	Page 6 (section 2.4 data collection)
4. Gender	Was the researcher male or female?	Page 6 (section 2.4 data collection)
5. Experience and training	What experience or training did the researcher have?	Page 6 (section 2.4 data collection)
Relationship with participants		
6. Relationship established	Was a relationship established prior to study commencement?	Yes, inferred from page 6
7. Participant knowledge of the interviewer	What did the participants know about the researcher? e.g. personal goals, reasons for doing the research	Inferred from pages 6 and 8 (Data collection and analysis sections)
8. Interviewer characteristics	What characteristics were reported about the interviewer/facilitator? e.g. Bias, assumptions, reasons and interests in the research topic	Reason for conducting the research (see aim of study in pages 4 (study design) and 6 (data collection) of manuscript)

**Domain 2: study design**		
Theoretical framework		
9. Methodological orientation and Theory	What methodological orientation was stated to underpin the study? e.g. grounded theory, discourse analysis, ethnography, phenomenology, content analysis	Pages 5, 6 and 8 (section 2.3 conceptual frameworks, and sections 2.4 and 2.8 i.e. data collection and analysis).
Participant selection		
10. Sampling	How were participants selected? e.g. purposive, convenience, consecutive, snowball	Page 6 (section 2.4 sampling and data collection, and section 2.5 definition of stakeholders)
11. Method of approach	How were participants approached? e.g. face-to-face, telephone, mail, email	Face-to-face: Page 6 (section 2.5 definition of stakeholders)
12. Sample size	How many participants were in the study?	Page 6 (section 2.4 sampling and data collection)
13. Non-participation	How many people refused to participate or dropped out? Reasons?	Page 6: not provided
Setting		
14. Setting of data collection	Where was the data collected? e.g. home, clinic, workplace	Page 6 (section 2.4 data collection)
15. Presence of non-participants	Was anyone else present besides the participants and researchers?	Not applicable
16. Description of sample	What are the important characteristics of the sample? e.g. demographic data, date	Page 6 and 8 (section 2.4 on data collection and section 4.1 on characteristics of stakeholders)
Data collection		
17. Interview guide	Were questions, prompts, guides provided by the authors? Was it pilot tested?	Additional file 2 and page 6
18. Repeat interviews	Were repeat interviews carried out? If yes, how many?	Not for this legacy evaluation
19. Audio/visual recording	Did the research use audio or visual recording to collect the data?	Page 6 (section 2.4 data collection)

20. Field notes	Were field notes made during and/or after the interview or focus group?	Yes.
21. Duration	What was the duration of the interviews or focus group?	Page 6 (section 2.4 data collection)
22. Data saturation	Was data saturation discussed?	No, Not applicable
23. Transcripts returned	Were transcripts returned to participants for comment and/or correction?	No, Not applicable.
**Domain 3: analysis and findings**		
Data analysis		
24. Number of data coders	How many data coders coded the data?	Page 8 (section 2.8 data analysis)
25. Description of the coding tree	Did authors provide a description of the coding tree?	Page 8 (section 2.8 data analysis)
26. Derivation of themes	Were themes identified in advance or derived from the data?	Page 8 Mainly derived from the data
27. Software	What software, if applicable, was used to manage the data?	Page 8
28. Participant checking	Did participants provide feedback on the findings?	No
Reporting		
29. Quotations presented	Were participant quotations presented to illustrate the themes/findings? Was each quotation identified? e.g. participant number	Yes, Page 10 to 13
30. Data and findings consistent	Was there consistency between the data presented and the findings?	Yes, there was.<br>Page 9 to 13
31. Clarity of major themes	Were major themes clearly presented in the findings?	Yes. they were.<br>From page 9 to 13
32. Clarity of minor themes	Is there a description of diverse cases or discussion of minor themes?	Discussion of major and minor themes<br>From page 9 to 13
