# Supplementary material for: Sustainability of the Effects and Impacts of Using Digital Technology to Extend Maternal Health Services to Rural and Hard-to-Reach Populations: Experience From Southwest Nigeria
Source: Front Glob Womens Health. 2022 Feb 8;3:696529. doi: 10.3389/fgwh.2022.696529 (PMC8861509; doi:10.3389/fgwh.2022.696529)
Supplement: Supplementary file 2 [file Data_Sheet_2.PDF]

Appendix 3: Story Collection Forms for Most Significant Changes

Health Workers and Facility Managers

**Name of Interviewer:**	
**Date & Time of interview:**	
**Respondent ID/Code:**	
**Age:**	
**Gender:**	
**Village/LGA:**	

Questions:

Do not give stakeholders clues about what you may want to hear. The story should be about their ideas about change, not yours. Try to avoid producing 'expected' accounts of change.

**1.** ***In your opinion, what is the most significant change that has occurred in the IPP Nigeria programme especially over the last 12 months?***<br><br>**Probe:** Ask stakeholders to tell their story in following three stages:
The situation **<u>before</u> participating in IPP Nigeria:**
Significant change(s) observed/experienced **<u>during IPP Nigeria</u>:**
Change(s) observed/experienced **<u>in the last 12 months</u>:**
In your opinion, why is this the most significant change?
2. Are there any other change(s) you observed/experienced in the past 12 months? If yes, please tell us about the change(s)
**Domains of change to probe with this stakeholder group:**<br>1) Standard of healthcare provision associated with VTR and CliniPAK<br>2) Ongoing use of clinical videos and CliniPAK in the last 12 months<br>3) Staff attitude towards patients
Probe the above domains with stakeholders who don't mention these in their initial answer to question 1:<br>1) **Standard of care:**<br>In your opinion, what are the most significant changes in past 1 Year (Positive and negative changes) in standard of service provision in this health facility?

2) Ongoing use of high-quality videos and CliniPAK:

In your opinion, what are the most significant changes in past 1 Year (Positive and negative changes) in the use of clinical videos and CliniPAK for providing life-saving care in this facility?

3) Staff attitude towards patients

In your opinion, what are the most significant changes in past 1 Year (Positive and negative changes) regarding attitude of staff towards patients in this facility?

<u>Story Collection Form for Service Users</u>

**Name of Interviewer:**	
**Date & Time of interview:**	
**Respondent ID/Code:**	
**Age:**	
**Gender:**	
**Village/LGA:**	

Questions:

Do not give stakeholders clues about what you may want to hear. The story should be about their ideas about change, not yours. Try to avoid producing 'expected' accounts of change.

**1.** ***In your opinion, what is the most significant change that has occurred <u>in your life</u> in the last 12 months following participation in IPP Nigeria programme?***<br><br>**Probe:** Ask stakeholders to tell their story in following three stages:
The situation **<u>before</u> participating in IPP Nigeria:**
Significant change(s) observed/experienced **<u>during IPP Nigeria</u>:**
Change(s) observed/experienced **<u>in the last 12 months</u>:**
In your opinion, why is this the most significant change?
2. Are there any other change(s) you observed/experienced in the past 12 months? If yes, please tell us about the change(s)
**Domains of change to probe with this stakeholder group:**<br>1) Standard of healthcare provision in this facility<br>2) Changes in your life linked to participation in IPP Nigeria project<br>3) Staff attitude towards patients
Probe the above domains with stakeholders who don't mention these in their initial answer to question 1:<br>**1) Standard of care:**<br>In your opinion, what are the most significant changes in past 1 Year (Positive and negative changes) in standard of service provision in this health facility?<br>**2) Staff attitude towards patients**<br>In your opinion, what are the most significant changes in past 1 Year (Positive and negative changes) regarding attitude of staff towards patients in this facility?

Story Collection Form for Community Members

**Name of Interviewer:**	
**Date & Time of interview:**	
**Respondent ID/Code:**	
**Age:**	
**Gender:**	
**Village/LGA:**	

Questions:

Do not give stakeholders clues about what you may want to hear. The story should be about their ideas about change, not yours. Try to avoid producing ‘expected’ accounts of change.

**1.** ***In your opinion, what is the most significant change that you have observed <u>in this facility</u> in the last 12 months as part of the IPP Nigeria programme?***<br><br>**Probe:** Ask stakeholders to tell their story in following three stages:
The situation **<u>before</u> participating in IPP Nigeria:**
Significant change(s) observed/experienced **<u>during IPP Nigeria</u>:**
Change(s) observed/experienced **<u>in the last 12 months</u>:**
In your opinion, why is this the most significant change?
2. ***Are there any other change(s) you observed/experienced in the past 12 months? If yes, please tell us about the change(s)***
**Domains of change to probe with this stakeholder group:**<br>**1)** Standard of healthcare provision in this facility<br>**2)** Staff attitude towards patients
Probe the above domains with stakeholders who don’t mention these in their initial answer to question 1:<br><br>**1) Standard of care:**<br>In your opinion, what are the most significant changes that you have observed in past 1 Year in standard of service provision in this health facility?<br><br>**2) Staff attitude towards patients**<br>In your opinion, what are the most significant changes in past 1 Year (Positive and negative changes) regarding attitude of staff towards patients in this facility?

Story Collection Form for Policymakers

**Name of Interviewer:**	
**Date & Time of interview:**	
**Respondent ID/Code:**	
**Age:**	
**Gender:**	
**Village/LGA:**	

Questions:

Do not give stakeholders clues about what you may want to hear. The story should be about their ideas about change, not yours. Try to avoid producing 'expected' accounts of change.

**1. *In your opinion, what is the most significant change that has occurred in the IPP Nigeria programme especially over the last 12 months?*** <br>**Probe:** Ask stakeholders to tell their story in following three stages:
The situation **before** **participating in IPP Nigeria:**
Significant change(s) observed/experienced **during IPP Nigeria**:
Change(s) observed/experienced **in the last 12 months**:
In your opinion, why is this the most significant change?
2. Are there any other change(s) you observed/experienced in the past 12 months? If yes, please tell us about the change(s)
**Domains of change to probe with this stakeholder group:**   **1)** Government/other stakeholders funding of the project   **2)** Ongoing use of clinical videos and CliniPAK in the last 12 months   **3)** Unexpected outcomes and impact
Probe the above domains with stakeholders who don't mention these in their initial answer to question 1:   **1) Government (and other stakeholders) funding of the project:**   In your opinion, what are the most significant changes in past 1 Year (Positive and negative changes) regarding government/other stakeholders funding the IPP Nigeria project? <br>**2) Ongoing use of high-quality videos and CliniPAK:**   In your opinion, what are the most significant changes in past 1 Year (Positive and negative changes) in the use of eHealth tools for providing life-saving care? <br>**3) Unexpected outcomes and impact**   In your opinion, have you observed any significant changes in past 1 Year (Positive and negative changes) that you didn't expect at all?
