# Supplementary material for: Sustainability of the Effects and Impacts of Using Digital Technology to Extend Maternal Health Services to Rural and Hard-to-Reach Populations: Experience From Southwest Nigeria
Source: Front Glob Womens Health. 2022 Feb 8;3:696529. doi: 10.3389/fgwh.2022.696529 (PMC8861509; doi:10.3389/fgwh.2022.696529)
Supplement: Supplementary file 3 [file Data_Sheet_3.PDF]

Appendix 1: Participant consent form

**Consent to take part in the study of:**<br><br>**Extending Health Services to remote areas in Nigeria using Satellite Communication to strengthen health systems and improve health outcomes (EXTEND Project)**	Add your initials or thumb print next to the statements below if you agree
I confirm that I have read and understand the information sheet dated 1st June 2017 explaining the above research project and I have had the opportunity to ask questions about the project.	
I understand that my participation is voluntary and that I am free to withdraw at any time before or durin g the interviews without giving any reason and without there being any negative consequences. In addition, should I not wish to answer any particular question or questions, I am free to decline.<br><br>Contact number of lead researcher is: +44 780 150 6584<br><br>I understand that any data/responses already provided will be deleted. I also understand that participants can withdraw their data up to 48 hrs after the individual interview, after which time data analysis will have begun.	
I understand that the interviews may be audio-recorded. I give permission for members of the research team to make audio-recordings of the discussions.	
I give permission for members of the research team to have access to my anonymised responses. I understand that my name will not be linked with the research materials, and I will not be identified or identifiable in the report or reports that result from the research.<br><br>I understand that my responses will be kept strictly confidential.	
I agree for the data collected from me to be stored and used in relevant future research in an anonymised form.<br>I understand that the results of the study will be published in academic journals. I agree that direct quotations from my responses can be published in anonymised form as part of illustrating findings and interpretation of the study.	
I agree to take part in the above research project and will inform the lead researcher should my contact details change.	

Name of participant	
Participant's signature or thumb print	
Date	
Name of person taking consent	
Signature	
Date*	

*To be signed and dated in the presence of the participant.

Once this has been signed by all parties the participant should receive a copy of the signed and dated participant consent form, the letter/ pre-written script/ information sheet and any other written information provided to the participants. A copy of the signed and dated consent form should be kept with the project's main documents which must be kept in a secure location.
