# Supplementary material for: Sustainability of the Effects and Impacts of Using Digital Technology to Extend Maternal Health Services to Rural and Hard-to-Reach Populations: Experience From Southwest Nigeria
Source: Front Glob Womens Health. 2022 Feb 8;3:696529. doi: 10.3389/fgwh.2022.696529 (PMC8861509; doi:10.3389/fgwh.2022.696529)
Supplement: Supplementary file 4 [file Data_Sheet_4.PDF]

Appendices: Ranking of Most significant change stories

Appendix 1: Quality of care

Kindly rank in order of significance, from 1 to 3; with 3 being the Most Significant story and 1 being the least significant story

N.B: Pseudo names have been used to de-identify the participants

Serial Number	Significant story	Ranking
		Univ of Lagos
1.	Chief Johnson is a 75 years old man and the chairman of the ward health association of the community. He leads the community association to perform the role of an intermediary between the health facility and the community. He also was responsible for teaming up with health personnel to purchase drugs needed by the community. He shares his story of most significant change in the past 12 months.<br><br>Chief Johnson believed that the quality of care has been consistently good in the community considering the quality of health workers available. He believes that since nobody died while receiving care, they quality of care is good. Commenting on the quality of care, he said "it has been going well, no one has died here. Pregnant women come and they receive drugs, deliver babies and go back home well" He is also of the opinion that clients would normally report erring health workers if they display poor attitude and that nobody has reported any health workers in the past 12 months. According to him, "*They don't treat them badly, there is no one that has come to report that they came here and they were not given drugs, either pregnant women or sick patients, they have not come to report that*".	1
2.	Chief Okana Mosa is a 70 years old man and the chairman of the ward health committee of one of the control communities in Ondo State. He was born in the community and had lived in the community and he and his family members patronise the health facility. He has the responsibility of representing the community in any matters regarding health. He tells his most significant stories on the standard of health care<br><br>Chief Okana Mosa believed the most significant change witnessed in the past 12 months is the improved standard of care for the people. Chief Mosa stated he observed changes in the care of their people when he declared that the "*care of our pregnant women and children is our main concern because if we are not doing well the community will be complaining that their health facility is not doing well and they won't want to come here……*". According	1

	to him, the quality of care has improved in the area of drugs being administered because funds to purchase drugs is now released regularly by the government. In his words, he said "*This period we have started seeing the hand of the government little by little, like them giving us little money, then we use it to get more drugs that the pregnant women need"*. He added that availability of drugs has built confidence in the people and gives them hope that they will be well treated and will not be asked to go and buy drugs when they come to the facility. According to him, "*So when they come we don't tell them to go outside and get drugs, we now give them drugs except a few things that might be needed"*.	
3.	Mrs. Alice is 39 years old and practices as a community health technician in one of the control sites of the IPP Nigeria project. She has been working in the facility for more than 2 years. She performs delivery and is in charge of routine immunization and also administers injection to clients. She shuttles between Ondo town, where she lives and the community where she works on a weekly basis. She tells her most significant change story.<br><br>Mrs. Alice tells her story around the quality of care which she believes has improved significantly. According to her, *"In the last one year, then when I just got here there was money, some of the PBF [Performance Based Funding] you know they do give us money. The money was surplus, so we had enough to do demonstration, we do handkerchief and rubber, and we give people who just put to bed"*. Because the quality of care improved as a result of the PBF, the number of clients patronizing the facility improved. In her words, *"it also makes more people come here because sometimes we can make free tea for them and give them some few things… "then all our antenatal days we do demonstration and we can cook eggs, beans, anything that will not cost us too much money."* Another development that positively affected the quality of care was the 24 hours service introduced into the facility which attracted people from other neighbouring communities to patronise the facility. According to Mrs. Alice *"their health center was not functioning well, their staffs do not sleep over like we do, we work 24 hours and there is no time you come here that you won't meet us. So their pregnant women do come to our facility for delivery"*. But now, everything is gradually changing, because the PBF will soon be over and the facility is reducing its expenditure on incentives that bring clients to the facility. Mrs Alice stated that previously their patients do not have to pay to access care or buy drugs in their facility but now the patients have to pay for some drugs or buy others things that are not available in the facility. Explaining what is the situation, Mrs. Alice said, *"but now we don't have much like before. So now we have to be calling people, you know then it was free drugs but now not everything is free. It's the children that get free drugs and not adults, and some of the children drugs that are very expensive and we don't have it, they will go and buy"*	2

4.	Mrs. Buba is a 25 years old service user and mother of 2 children who have been using the facility for more than one year. She tells her significant story on how the intervention has improved the quality of care they received from the facility.<br><br>Mrs Buba stated that the use of the VTR device has been of significant help to her because they learn a lot from watching some of the videos on the device. According to her, *"On clinic days when we come and we finish singing and exercise they will tell us to watch the pregnant women, the type of food they dish for them and how they give themselves rest or if they are having pains in their tummy, how they quickly rush down to the clinic"*. Mrs. Buba recounted how she had benefited from watching video because it has helped her to gain a lot about things she did not know previously especially about hygiene and eating good food. In her words, "*You know we are in the village and we act like village people, when we watch it we see how we can change, like how we can take care of our home even if we are using one room, if we are using it in a dirty manner we should change because of our health"* She further mentioned how it has positively changed her pattern of diet. *"And the food we eat, because at times we eat only fufu, but they let us know that we are not meant to eat just fufu"*. However, she confirmed that the last time she saw the device being use in the facility was when she came to immunize her baby about 6 months ago. She doesn't know whether the device is still being used anymore or not.	3
5.	This story was told by Mr. Papu, the chairman of the community health association in one of the control sites of the IPP Project. Mr. Papu and his committee members are responsible for overseeing and managing health related issues affecting the community. They serve as the link between the community and health workers. He has been the chairman of the community health association since 2014. He told us about the significant change that he felt has taken place in the health of the community during the past 12 months.<br><br>Mr. Papu described how the Performance Based Funding (PBF) programme of the World Bank has enhanced quality of care in the community. He mentioned that the PBF really closed some gaps in the quality of care being provided, because prior to the funding, drugs were not supplied base on community needs. According to him, *"we are given some drugs by the government, then PBF gives us money to buy drugs by ourselves in Akure and we bring it here. Before then it was the government that gives us drugs, they didn't know some of the drugs we needed, and that time when they bring us drugs it will expire because that is not what we need but now they don't expire again because we know the nature of sickness common here."* Availability of relevant drugs through the PBF has largely contributed to the quality and standard of care in the facility. Now, drugs don't get expired as before, pregnant women no longer need to buy prescribed drugs and according to Mr. Papu, *"since we now have money to buy drugs, we have it inside and bring it out when needed, it is better than before"*.	2

6.	Mrs. Aku is a 49 years old nurse and the facility head in one of the health centre. The health facility is one of the intervention facilities on the IPP Nigeria Project. She supervises all the work that is being carried out in the facility at all the scales including delivery, immunization, administering drug to patients, then mobilizing community to inform them about any other program. She is in charge of the everyday life of the facility. She tells her significant change story.<br><br>Mrs Aku believes that the standard of care a improved with the introduction of the IPP Nigeria project. Because, according to Mrs. Aku, "*we gained more knowledge; personally I have more knowledge whenever I watch the video. You know we are supposed to refresh our memory. So that VTR when you are watching it, you will be learning more"*. Mrs. Aku recounted how the use of VTR has helped them to know they were applying archaic method of delivery and to help them improve on their delivery skills. In her words, *"before we don't use partograph for taking delivery but from watching video on VTR they taught us how to use partograph, how to chart it, the time you are supposed to check your patient, the time you are supposed to enter the chart if the patient is not up to 4 centimetre we should not touch the partograph. So later when I have the knowledge I told all my colleagues that this is how [we are going to be taking delivery]"*. So, they applied the method on one of their patients who have been having repeated stillbirth; the woman have had up to 2 or 3 still births. When she presented at the facility, the partograph procedure was used for her and the delivery was successful. Mrs. Aku story went thus, "*One of our patients who usually experience stillbirth came to the facility to deliver, usually, her baby will not cry at birth and the baby will eventually die. But this time we used the partograph to monitor her delivery. So it was the partograph that we used to monitor her delivery and the baby is up to two years now"*. This is one of the skills learnt by health workers while using the VTR and it has certainly impacted positively on the quality of care in the facility even after the project ended.	3
7.	Stella is a 26 years old female health worker and the Deputy Facility Head of a community Health Facility. She has been working in the facility for nearly a year assisting the facility in most her duties, especially when she is not around. Due to shift schedule mostly oversee activities of the facility in the afternoons and evening hours. She tells her story of most significant change of how the project has impacted on quality of care.<br><br>Stella believed that standard of care in the past 12 months have also changed, especially changes in delivery care. She told a story how a pregnant woman about to delivered was rushed to the facility *"the woman was stressed; the baby had stayed long. Later when we delivered the baby we were like hey will this baby die?... Maybe we should take this baby away. So one of us now said "ha there is one thing that they taught us about letting baby to*	3

	cry", so we now used it, so, we applied err what is called, manual oxygen for the baby. Later the cord was like it is not shaking, we now said *"ha this baby will not cry oh, even the mother of the baby was crying saying that "will they throw her child away?"* So with the help of that manual oxygen the baby survived. We did it know that it can work like that. On what should be done to improve standard of care, Stella was quick to add that drugs was a major problem in the facility. According to her *"if they can be giving us free drugs, you know some people, all they want is give me free injection, I think if we have drugs, that will be better".*	
8.	Esther is a 49 years old nurse and heads the head facility in a small town in the suburb of Akure. She has been the head of the facility for up to 6 years. Her job is to oversee the daily activities of the facility and to supervise other health workers. Esther did not know anything about IPP Nigeria Project but could relate to us some of the significant changes that took place in the facility during the past 12 months.<br><br>According to her, one of the most significant changes observed was that the number of people patronising the facility has increased. In her words, *"when we look at our daily performance like OPD we are increasing in number, when you talk about immunization people obtain immunization they are very very ok and [they are] increasing every time, every month".* The main factor responsible for this was that the facility runs a 24 hours service which would make clients to come any time of the day and night. Again, the facility was able to hire health staff on its own and provided accommodation for them within the facility. Buttressing this further, she declared, "*we are always here 24/7, we have contract staff which were not employed by the local government but through this program I am able to accommodate, to employ contract staff. At any time, 24/7 we have staff that maintain this clinic for us".* This significant change was linked to the Performance Based Funding (PBF) programme of the World Bank which enabled the facility to access funds based on performance. Through the PBF, drugs were made available free for pregnant women and under five children but subsidized for adults.	2
9.	Anita is a policy maker in Ondo State. She oversees programme planning and data capturing and reporting of health issues in the primary health care of Ondo State. She tells of the most significant change stories resulting from the IPP Nigeria project and how the project has impacted on standard of care.<br><br>One area where significant change was recorded was in the standard of care provided by health workers. Anita recounted how semi-skilled health workers aided a delivery with the help of what was learn on the VTR. According to her, a pregnant woman reported at the facility when the facility head was not available. The health worker was confused, not knowing what to do. When we asked him how he felt he said he was confused saying *"what do I do at this point since I'm the only one available here?". "How do I help this woman so that the baby and the woman will survive?"* But, from what he had learnt from the VTR he made up his mind to take the	3

	delivery, so, according to Anita *"he was following it step by step. He performed the, you know, procedure and until he was able to deliver that baby"*. The health worker had not really done anything like that before but he was empowered by what he learnt from the VTR. So, the story was told and the success caught the attention of people. So, according to Anita, *"there was a report that was compiled one time and the mother was visited. The child is doing very well and also the mother is doing very well"*. Anita could not hide her joy about how the standard of care had improved as a result of the use of VTR. She said *"you know, when you see those things, it gladdens one's heart"*. According to her, the story told is to show that the VTR has improved the standard of care among health workers *"that VTR is really beneficial and it's really worth it…. it actually helps to improve their skills which invariably translated to improvement in quality of service"*. When asked about the effect the non use-age of the VTR in the past 12 months on the health system, she said "that can be known from the health workers' perspective, they might say *"Okay, we are not able to train using the VTR, we got a new staff now and we were supposed to put the new staff through, but the VTR was helping us before, now it's no longer available"*. She further emphasised on the importance of the VTR when she said *"It's something that any government should actually lay emphasis on, particularly at the PHC level where we have gross shortage of skilled manpower. But it's really so sad, it's no longer in use"*. Based on its usefulness and the impact the VTR has had on quality of service, Anita discussed the efforts of the State government made at securing a World Bank grants to scale it up. *"It is one of the things that we really would have loved to scale up, we made efforts, proposals have been written to scale it up but it was not approved for us"*.	
10.	Mr. Cole is a coordinator of Primary Health in one of the local government areas in Ondo State. As the primary health coordinator, he oversees health facilities and also conduct clinical services including immunization, family planning, malaria programs and maternal and child health. Mr. Cole has been engaged with the sector for more than 10 years. He shares his experience on the IPP Nigeria project during the phase it was implemented and what he considers as the most significant changes 12 months after the project has ended.<br><br>When the IPP Nigeria project started, it changed many things in health care deliver. According to him, *"generally it actually improved our services and when the project was on it allows the health workers that are involved to learn a lot of things about services generally and our data also improved during that period, so basically it has had, it actually affects the health positively, I mean health services positively. Well it was not as significant, it wasn't at the level it eventually was because the truth of the matter is that when the VTR was actually introduced because they get to learn about a lot of things through that project, it actually enhanced services and the output as well as the outcome of their service was greatly improved because before the introduction of the VTR the service was not at the optimum*". One specific area in which the IPP Nigeria project brought changes was in the	3

	number of deliveries and client patronage recorded at the health facility. According to Mr.Cole "*in terms of delivery, the deliveries actually improved. Also in terms of patronage because the VTR actually helped the health workers to be at optimal level, they learnt a lot of things through the VTR and that means if you are visiting a health facility and you are getting the services that you know, people go around to preach the gospel saying, "that facility is good, they are doing a lot of things there and they won't take your time"... So it actually improves the services"* At Awo Abode health facility, Mr.Cole recounted his experience of health worker's use of VTR and the outcome on the health facility. *"There is this facility at Awo Abode, before the introduction of the VTR and everything people were not actually coming there but because they saw all the physical things they brought in, they installed VTR there. This actually helps the community and I mean the people living in the community, they wanted to know what that thing is about, so they started coming there. So in the process of coming to that facility and the gospel spread within the community and eventually the head of the facility told me that the number of people coming for services has greatly improved. I even saw it on the data like now that place they don't usually have delivery before, they started taking delivery and it improved, things actually improved".*	
11.	Mrs. Phoebe is a senior nurse and midwife who double as the Facility Head of one of the comprehensive health centres which was selected as a control site. She is also a Deputy PHC coordinator of the local government area. Mrs. Phoebe has been working in the local government area for 25 years and understands administrative issues pertaining to health system and the community at large. She tells her most significant change story spanning past 12 months<br><br>The most significant change recorded in the comprehensive health centre was the large attendance the facility is recording during antenatal care and other minor health issues. For example, last January the facility recorded up to 100 antenatal patients. Mrs. Phoebe responding to why there was a surge in patronage she said *"Had it been we are not attending to them very well they will not be coming here unlike before. So we have more people in the morning shift to do the work, to make it convenient for us and also the clients"* What this meant was that there was a reorganisation of health personnel which re-distributed most of their health workers into morning shift and then a few into afternoon/night shift. This approach became necessary because of shortage of health personnel. Since most clients tend to patronise the facility in the morning hours, which also means more work on issues around data, the rational thing to do was to deploy more personnel to handle morning shift. The approach seems to be working well for Mrs. Phoebe because, according to her, *"when you get to the hospital and immediately they attend to you…. So they prefer to attend this place but some hospital you will sit down for 30 minutes to 1 hour, nobody will say madam what did come for, imagine something like that. So I think it is the one that is helping us"* Another important factor which Mrs. Phoebe thought was helping them to achieve this is that most health	3

	workers are indigenes of the community. According to her, *"you know we are from this place, myself I'm from this place and almost everybody, all of our staffs here we are from this place so this people are our people, we cannot fight them, we use to attend to them the way we are supposed to attend to them"* So, the close affinity regulates the attitudes of health workers and the attention they give to their clients. As Mrs. Phoebe puts it *"they know my family, they know my daddy, they know my brother, so if I don't attend to them they will say ha so so so person's daughter…. So we know our self very well"*. Another factor that might be driven the significant change is the prompt supply of drugs. Mrs. Phoebe declared *"The facility is receiving funds from N-SHIP (Nigerian State Health Investment Project) which is a World Bank Project and the funds are meant for purchasing drugs… they gave us money to buy drugs, so and we are using the money to buy drugs. They said we should be doing drug revolving something so we buy the drug, sell it to the patient in a small amount, so we are able to refund the money to the account"*	
12.	Mrs. Phoebe is a senior nurse and midwife who double as the Facility Head of one of he comprehensive health centres which was selected as a control site. She is also a Deputy PHC coordinator of the local government area. Mrs. Phoebe has been working in the local government area for 25 years and understands administrative issues pertaining to health system and the community at large. She tells her most significant change story spanning past 12 months<br><br>The re-distribution of health personnel also impacted on data quality in the past 12 months. Most clients patronise the facility during the morning hours which means that a lot of time would be devoted to enter their data. Data was usually captured manually. But because the facility was short staffed, they constantly experience some challenges with their data. According to Mrs. Phoebe "*before we were not using that idea and so it was giving us tough time. In terms of data we don't even meet up with data at all but since we have been practicing the way we are doing now, it is doing us a lot of good"*. Allocating more staff to work during the morning hours enabled them to *"have more people in the morning shift to handle the program; immunization, antenatal, all the discharge monitor the rotation aspect, every aspect, HIV so we have people on them that are taking the data… but during the time we were still doing afternoon, morning and night we don't have err staff to do all of that and so it is giving us a lot of challenge"* Now the facility has been able to cope with the number of clients patronising them. However, data was being captured manually.	2

Appendix 2: Facility patronage

<u>Kindly rank in order of significance, from 1 to 3; with 3 being the Most Significant story and 1 being the least significant story, the following stories on staff attitude</u>

N.B: Pseudo names have been used to de-identify the participants

Serial Number	Significant story	University of Lagos ranking
1.	This story was told by Mr. Papu, the chairman of the community health association in one of the control sites of the IPP Project. Mr. Papu and his committee members are responsible for overseeing and managing health related issues affecting the community. They serve as the link between the community and health workers. He has been the chairman of the community health association since 2014. He told us about the significant change that he felt has taken place in the health of the community during the past 12 months. According to him one of the significant changes recorded in the community is that patronage of the health facility became very low. According to him, Owena health facility was deemed as better so community members from Owena gada usually patronise the health facility at Owena Aiyetoro because health workers at Owena gada were not punctual. But recently, a new king at Owena Gada has instructed his people to stop using our health facility, so the patronage is very low. According to him, at Owena Gada, "*their staff [health personnel] do not sleep in their health facility, unlike our own, so most of the pregnant people, and those who are sick, used to patronise us. But suddenly they stopped it that this is another local government and people must not come here. It made those patronizing this place low than before"*	1
2.	Chief Johnson is a 75 years old man and the chairman of the community health association of Aseigbo community. He leads the community association to perform the role of an intermediary between the health facility and the community. He also was responsible for teaming up with health personnel to purchase drugs needed by the community. He shares his story of most significant change in the past 12 months.<br><br>Chief Johnson was not aware that any device was given to the facility and has not witnessed the use of the project's devices in the facility, not least the IPP Nigeria project. His response to the question on the use of the devise was that of a surprise. He exclaimed and said "Ha I have not seen that here". However, Chief, Johnson felt that there has bee some significant changes the facility has experienced in the last 12 months. According there has be a significant in the	1

	community patronage of the facility. In his words, *"Before we don't see people to attend to here but now people deliver here and when they are not feeling fine they get treated, we thank God for that. If someone sustains an injury at the farm they also take care of them, so the progress is moving forward…."* When asked what he thought was the reason for the increase in patronage, he was quick to refer to availability of drugs. According to him, the facility now boasts of sufficient drugs for their patients as a result of the improved collaboration between the CHA and health facility. The Drug Revolving Fund has enabled the CHA to be involved in health expenditure, particularly in the purchase of drugs. Chief Johnson described the situation around drug availability at the facility *"At times there won't be drugs in this place,.. when I go they will say they have approved funds for us then I will go there sign it and go collect the money at the bank. We will collect it and buy the drugs, if they come to call me I will come and see it with my eyes and I am happy".* Chief, Johnson also felt that the provision of a deep well was also a factor in the increase in the rate of patronage at the facility. Referring to the community efforts that produced the well, Mr. Johnson said, "they approved a well for us, that is what we dug over there".	
3.	Mrs. Alice is 39 years old and practices as a community health technician in one of the control sites of the IPP Nigeria project. She has been working in the facility for more than 2 years. She performs delivery and is in charge of routine immunization and also administers injection to clients. She shuttles between Ondo town, where she lives and the community where she works on a weekly basis. She tells her most significant change story.<br><br>Mrs. Alice tells her story around the quality of care which she believes has improved significantly. According to her, "In the last one year, then when I just got here there was money, some of the PBF [Performance Based Funding] you know they do give us money. The money was surplus, so we had enough to do demonstration, we do handkerchief and rubber, and we give people who just put to bed". Because the quality of care improved as a result of the PBF, the number of clients patronizing the facility improved. In her words, *"it also makes more people come here because sometimes we can make free tea for them and give them some few things… "then all our antenatal days we do demonstration and we can cook eggs, beans, anything that will not cost us too much money."* Another development that positively affected the quality of care was the 24 hours service introduced into the facility which attracted people from other neighbouring communities to patronise the facility. According to Mrs. Alice *"their health center was not functioning well, their staffs do not sleep over like we do, we work 24 hours and there is no time you come here that you won't meet us. So their pregnant women do come to our facility for delivery".* But now, everything is gradually changing, because the PBF will soon be over and the facility is reducing its expenditure on incentives that bring clients to the facility. Mrs Alice stated that previously their patients do not have to pay to access care or buy drugs in their facility but now the patients have to pay for some drugs or buy others things that are not available in the facility. Explaining what is the situation, Mrs. Alice	1

	said, *"but now we don't have much like before. So now we have to be calling people, you know then it was free drugs but now not everything is free. It's the children that get free drugs and not adults, and some of the children drugs that are very expensive and we don't have it, they will go and buy"* Mrs Alice expressed her worries that in the last one year that there has been reduction in funding to the facility. The government's funding partners have withdrawn some of the monies previously given to health facilities in Ondo State to provide free health care for their people. She also expressed her concern about their neighbouring community (Aiyetoro) that usually patronize them who no longer do so because their community head instructed them to start patronizing the own health facility. She explains further "*before, the people from Aiyetoro are always many here, they patronize us more than this Owena because there are a lot of quacks in Owena, those ones come very often. Then that their health center they said their health center is not that good, their staff are not very good but now their health center is now good, they have gone to report, so they have said those ones must not come here, that has also affected us. That is also one of my observation".*	

Appendix 3: Staff attitude

Serial Number	Significant story	University of Lagos ranking
1.	Mrs Clara is the health worker and facility head in one of intervention health facilities. She oversees every health care delivery services to the community by the centre and supervises health workers under working with her. She shared some interesting significant change stories on the positive impact the IPP Nigeria project had on staff attitude.<br><br>Exposure to the VTR did impact significantly on attitudes of health workers. *"Attitude is everything",* according to the head of facility because *"good behaviour and good attitude to patients can make more things to come, because if you are friendly to patient, more patients will come in, it is that patient that comes in that can help you collate more data. So it is that attitudinal change that can give us more things.*" She described how they all watched a video on the need for health workers to possess good attitude on the VTR and how it spurred deep and prolonged conversation among them. Mrs. Clara personally learnt that a leader could also display wrong attitudes to patient. She came away with an impression that *"not only the junior staff that can be misbehaving that even OIC can misbehave too! So it let's us know our attitude that we should change, we should be good and make good rapport with our patient.*" On how that has influenced the attitudes of staff under her, she has a story to tell: *"one of them complained of being indisposed. While she stayed outside the clinic, a patient came in to seek care. Immediately, she forgot about what was happening to her*	2

	and she stood up greeted and laughed. Though, I was planning to treat her because of the pain but that day, she forgot about what was doing her".	
2.	Chief Johnson is a 75 years old man and the chairman of the ward health association of the community. He leads the community association to perform the role of an intermediary between the health facility and the community. He also was responsible for teaming up with health personnel to purchase drugs needed by the community. He shares his story of most significant change in the past 12 months.<br><br>Chief Johnson believed that the quality of care has been consistently good in the community considering the quality of health workers available. He believes that since nobody died while receiving care, they quality of care is good. Commenting on the quality of care, he said *"it has been going well, no one has died here. Pregnant women come and they receive drugs, deliver babies and go back home well"* He is also of the opinion that clients would normally report erring health workers if they display poor attitude and that nobody has reported any health workers in the past 12 months. Chief Johnson has not observed any difference in the attitude of the health workers in the past 12 months. He stated that, just like before, the staff are treating the patients well and that everyone is happy. He added *"They don't treat them badly, there is no one that has come to report that they came here and they were not given drugs, either pregnant women or sick patients, they have not come to report that and fight with us"*.	1
3.	Mrs. Alice is 39 years old and practices as a community health technician in one of the control sites of the IPP Nigeria project. She has been working in the facility for more than 2 years. She performs delivery and is in charge of routine immunization and also administers injection to clients. She shuttles between Ondo town, where she lives and the community where she works on a weekly basis. She tells her most significant change story.<br><br>Mrs Alice hasn't noticed any change in their general attitude towards clients. There is an understanding between the community members and health workers that clients lodge a complaint about any unruly behaviour from either patients or facility staff to the facility head or Chairman of the WDC. This approach has helped maintain a cordial relationship between clients and health workers. She stated that their attitude towards their patients have been cordial and respectful and that they make every attempt to draw the patients closer and put smiles on their faces whenever they visit the facility for care. According to her, *"We are not cranky towards our patients, we draw them close to us, we make them laugh and all that, play with them and make jokes with them"*. But responding to whether there is any change in the attitude	2

	of health workers over the past 12 months, she said, "*No, it's the way we do that we still do. When staff notice that the patient is acting somehow to them, they report to the person in charge or the chairman (WDC) you called"***.**	
4.	This story was told by Mr. Papu, the chairman of the community health association in one of the control site of the IPP Project. Mr. Papu and his committee members are responsible for overseeing and managing health related issues affecting the community. They serve as the link between the community and health workers. He has been the chairman of the community health association since 2014. He told us about the significant change that he felt has taken place in the health of the community during the past 12 months.<br><br>Mr. Papu did not observe any significant change in the attitude of health workers in the past 12 months. In fact, in his words, *"there is no change because the staffs here, they are doing well"*. He pointed out the role of the leadership in coordinating other junior health workers: *"the person heading them is trying her best, she herself comes here to sleep and when the boss is hard working it will be difficult for the staff to be lazy. If the boss gets to work by 8 0'clock, what will the staff be doing that won't make her come early, but when the boss gets in by 12 then the staff will come by 10am*"	2
5.	Mrs. Aku is a 49 years old nurse and the facility head in one of the health centres. The health facility is one of the intervention facilities on the IPP Nigeria Project. She supervises all the work that is being carried out in the facility at all the scales including delivery, immunization, administering drug to patients, then mobilizing community to inform them about any other program. She is in charge of the everyday life of the facility. She tells her significant change story.<br><br>Mrs. Aku believes that an improvement in the attitude of her health workers was observed at the time the IPP Nigeria project was implemented. The VTR improved the rapport between clients and health workers. In this regard, she has this to say, "*even for my health workers here, the rapport with the patient before was not up to 100% but let's say it was 60% to the patient. But during that VTR when we watched it and then we do a meeting together, (we usually do staff meeting), the things that you learn you say it, I will say my own, then we now compile it together and use it to practice"* The VTR created a positive consciousness in the health workers as per how they need to relate with patients. According to Mrs. Aku, *"the VTR taught us how to have patience if at all that you, if any patients do something that is not good for you, so leave her. You will not fight at that moment but later call the patient and let her realize what she do that is not good"*. In her view, attitude of health workers towards the patients is very crucial to all other services being rendered. In her words, *"you know if we don't see patients we can't see any data to send, so patient is more important, so if the patients are coming we will send data, you know you cannot formulate or manufacture any"*.	2

6.	Stella is a 26 years old female health worker and the Deputy Facility Head. She has been working in the facility for nearly a year assisting the facility in most her duties, especially when she is not around. Due to shift schedule mostly oversee activities of the facility in the afternoons and evening hours. She tells her story of most significant change.<br><br>One area in which Stella thought there has been a change was in the attitude of health workers. In the past, clients were used to complaining about attitude of health workers but now all that has stopped. Health workers now have good disposition with their clients. According to Stella, "most of the times patients use to complain that nurses are harsh, they are this, they are that, but now we use to pet them so that they will be able to come to hospital and they won't be scared". Poor attitude displayed by health workers do keep client away from the facility. According to Stella "*some may not even come to this place they will be like "ha no oh, I don't want to come to health centre they are too harsh" but I think that as stopped now"*. The reason why the facility is experiencing this change, according to Stella was because the health workers with poor attitudes have been posted out. According to her, "*they have changed the workers here because those people are complaining that because of attitude they don't need to come. We wanted to know if Stella experienced a change in attitude, she said "my attitude did not change because I am very nice to them, so I use to pamper my patient and I am very sure that my patients will tell you that I like them"*.	2
7.	Esther is a 49 years old nurse and heads one of the control facility in a small town in the suburb of Akure. She has been the head of the facility for up to 6 years. Her job is to oversee the daily activities of the facility and to supervise other health workers. Esther did not know anything about IPP Nigeria Project but could relate to significant change on staff attitude.<br><br>Esther felt that health workers would only display poor attitude when they work in environment that is not conducive. According to her, if *"these contract staff we are paying them little amount, when we have bonus we are giving them, they are comfortable, the environment is comfortable for them. Now we build two rooms for them so that they are able to sleep and taking care of themselves"* But in terms of change in attitude in the past 12 months She said, "It is still the same attitude, because they are fearing that when this program end what is their fate, and I tell them "*don't worry we are going to pay your salary, just let's work together. If we work together we can achieve it, we can make it to pay the salary"*.	1

Appendix 4: Ongoing Use of e-health technology

Serial Number	Significant story	University of Lagos ranking
1.	Mrs Clara is the health worker and facility head in one of intervention health facilities. She oversees every health care delivery services to the community by the centre and supervises health workers under working with her. She shared some interesting significant change stories on the use of e-health technologies.<br><br>From her stories, we found that the introduction of CliniPAK significantly and positively altered data capturing, data quality and data transmission. Mrs. Clara described how CliniPAK helped them to improve on data capturing by dis-aggregating data into different components. According to her, *"before we were just collating data, to be sincere, but now it let us know specific data that is to be collated not just general data"*. Not being able to collect specific data did not happen because they did not have the tools to do so, but because they did not even know the types of data to collect. Mrs. Clara said further *"We don't know that before, we just enter data, we have general data"* She also told a story of how the CliniPAK helped them to organise their data collection process. So, there was a supervisory visit and data quality needed to be monitored, according to Mrs. Clara, *"they asked us to bring one card and then go to that data, they search for the name on the card, even we were very afraid that they may not see this name in this general OPD because we have many names there. We have OPD, that OPD will enter general OPD, family planning will enter general OPD, ANC will enter general OPD. But now they ask us to bring card, it is card they used to trace that general OPD data and we are very happy that day, they picked [randomly] from ANC and from others cards, they just used that card, they didn't take register. When they looked for it, they saw it and we were very happy that day*". But all that has changed as the facility has reverted to the manual/paper mode of data capturing because the CliniPAK is no longer in use. Mrs. Clara further provided insights into how the change is affecting them, *"we cannot send [data] with I-pad again that is it"*. Although she claimed that reverting to manual/paper mode has reduced the burden of work on health workers, the advantage of using CliniPAK far outweighs the burden that comes with using it. According to her, *"Although data is still being collected, it is no longer as comprehensive as before"*. The use of the CliniPAK did not only help in data capturing, it also help to build the IT capacity of health workers and enhanced their self-esteem. Health workers would apply themselves to studying the Ipad in order to understand how it works. Overtime, they were able to manipulate the tablet and use it to enhance the quality of services provided. According to Mrs. Clara, "*because it is a new thing, so we need an intelligent person that knows about computer so we have to apply ourselves, it made us to even know it by force.*	2

	It is just like computer, you have to ask question and answer it. So even that time, staff were not enough to enter all data on the register; family planning, ANC, HIV but everything they will ask is inside that I-pad. But what I know is it makes us improve, they make us know what we are doing in the health centre”.	
2.	Miss Helen is a junior community health extension worker assisting the facility head in one of the intervention communities. She is 27 years old and shuttles between Akure and her health facility on a weekly basis. According to her, “I come here on Monday, so we will sleep here from Monday to Friday, then on Friday I will go back to Akure” She did not participate in the training before the intervention started but she was trained by her colleagues and she has used it and understands how it works. She tells her most significant change story.<br><br>She recalls how they were managing and processing data before the introduction of the CliniPAK and the stress associated with reporting data manually. According to her, “*we will carry register to the basic health center to go and do validation and all the summary of the work is there” But once the CliniPAK was introduced the stress about having to enter data manually before attending to patient has reduced. Responding to this claim, she said “Since we have been using it (CliniPak device) we noticed that our work is different and there is no stress for us”* Apart from the improvement in data collection and management, Miss Helen also emphasised the advantaged derived from the use of the VTR in its usefulness in guiding health personnel in the process of care giving. According to her, *“What is most important for me is how it (VTR device) helps us to know the work we are doing and is guiding us, so if there is any mistake if we quickly check it, like removal of placenta if I have forgotten something, I will go and watch it and it will help me”* She stated that the most significant change they’ve experienced in the facility is the use of the VTR device, “*it teaches us some work like how to take delivery is on it. If we watch it in case there is anything, we can improve on we will know, there are different things there that help us with our work*”. She gave an example how the CliniPAK improve diagnosis and prescription for clients and sometimes correct any error in data input. According to her, “It has helped us because it asks some questions. If we enter their names, it will ask us if we have given this drug, which drug we haven’t given. We can forget to ask the patient that what drug have they used, but immediately we enter the patients name it will ask us and then we use that opportunity to ask”. However, she was concerned that the VTR device has not been functional for up to 6-7 months “*what is left to be worked on is like one year, let me say going to 6-7 months they locked it. All the things we watch that help us all the antenatal and postnatal videos they closed it and left only the one which we use to enter data and it did not assist us*”. She also added that their work has improved significantly as a result of the IPP Nigeria project and that because of that more clients are patronising the facility. In her words, “*because of all the things that were done, we started getting more patients”.* She emphasised that the process of data collection, transmission and validation has been made easier for them in the facility since the introduction of the CliniPak device.	2

3	Mrs. Aku is a 49 years old nurse and the facility head in one of the health centre. The health facility is one of the intervention facilities on the IPP Nigeria Project. She supervises all the work that is being carried out in the facility at all the scales including delivery, immunization, administering drug to patients, then mobilizing community to inform them about any other program. She is in charge of the everyday life of the facility. She tells her significant change story.<br><br>According to her, the use of the VTR was a major area where the IPP Nigeria brought significant change on the health care delivery among health personnel in the facility. She affirmed that before the introduction of CliniPAK, "there were many things that we do here that we didn't know how to do it, that is we were doing it wrongly. When we watched the VTR it taught us about some aspects from which we derive knowledge" She went on to tell of a story of the use of VTR has impacted on their delivery skills. According to her, *"before when we take delivery, we don't usually clean the baby like that, so we just cut the cord, then oil the baby, pack the baby and close the baby. But when we watch VTR so in that video we saw that when you deliver a baby you take the baby to the mother first, then you clean with dry cloth, then you wrap the baby. You never, you just cut the baby the cord immediately. Then after that, that baby will stimulate to breast err where the breast is and you can begin to suck the breast. Then after that when you cut the cord you now take the breast to the child. That is one particular thing that I derive from that video."* This procedure has helped them to scale up delivery skills and to upgrade quality of service to clients. The VTR has also helped in mobilizing community members to visit the facility and interact more with health workers. When pregnant women or community members watch the videos, they go into the community to inform others about the what they have learnt. According to nurse Aku, *"It [VTR] mobilize our community, even the patient began to patronize us because if any of them maybe they get pregnant and they don't want to register here, some of them will tell their neighbour "when we go for the clinic you will go and look at the video there", so that mobilized our pregnant women even our mothers"*. The use of VTR has also helped them to forge stronger relationships with women, most especially pregnant women. Mrs. Aku further explained "*There is changes because you know the people that are usually watching something when they come for the clinic, at times it may not be on clinic day, they say "ha aunty nurse, I have come to greet you, what of that our video?" Then we will just put it on the table and they will watch"*. Sadly, the facility has not been able to hold women and client together as before ever since the project stopped. According to her, "but since that thing has stopped, if they come and say "aunty that video, I still want to watch it oh", Then we tell them "sorry oh that thing is not working again", So now they feel somehow saying how can we give them something which they like and we now take it back from them. In essence, the strong rapport established between health workers and their client is now fractured since the stoppage of VTR.	2
4	Mrs. Aku is a 49 years old nurse and the facility head in one of the health centre. The health facility is one of the intervention facilities on the IPP Nigeria Project. She supervises all the work that is being carried out in the facility at	2

	all the scales including delivery, immunization, administering drug to patients, then mobilizing community to inform them about any other program. She is in charge of the everyday life of the facility. She tells her significant change story.<br><br>Her stories depict that the IPP Nigeria project brought significant change in the way in which routine health data was being collected and transmitted. Mrs. Aku recounted how CliniPAK improved the collection and transmission of data. In her words, *"It has helped us because by the time we have 4 patients in a day, I find that immediately we just enter the drug, the pharmacy, OPD, everything we do it on that tab, then the information will go directly to the board. But this time now they don't see all the information again in that board"* But before the introduction of the CliniPAK, Mrs. Aku stated that they used to carry all their *"registers to LGA for validation once in a month, so all the activity that we are carried out here. But that one once in a month, but when we started using the VTR, at times when we don't send out our data to the board, they will be calling me saying, "we have not seen your work for the day o".* So I will know that we are supposed to send that thing to them but now as that thing [VTR] has stopped now, nothing is working. The transmission of data is now mainly done using manual method and they have to wait for many months before they can do that. Describing how data is being transmitted now, Mrs. Aku exclaimed, "Ha when we are carrying that register sir, it is BAGCO bag that we are using oh (laughs) you know and the small register it is ANC register that is smaller, all these ones they are big big registers, it is not easy for us to be carrying all these, so if they can help us to do it like that, so it will help us sir<br><br>Mrs. Aku also believed that the use of the CliniPAK has empowered health workers significantly in the process of diagnosis and care. The tablet would diagnose client's condition accurately and provide a lead on the type of treatment to administer. When asked how the CliniPAK has empowered health workers, she exclaimed, *"Ha! It has empowered us oh! Because that thing is like computer, and when you are operating it, it will be directing you [on what to do]. It will request for the name of patient, the address, and everything. Then in terms of diagnosis it will direct you, with the complaint of the patient you will know the type of diagnosis that you supposed to give even to the drug in the pharmacy. And even if you suppose to refer the patient you will know that you suppose to refer the patient".* When asked if the CliniPAK is still being used, she replied, "since we stopped using the tablet, we have not been able to enjoy all of the benefits. We have gone back to our old ways of attending to patients".	
5.	Anita is a policy maker who oversees programme planning and data capturing and reporting of health issues in the primary health care of Ondo State. She tells of the most significant change stories resulting from the IPP Nigeria project.<br><br>The use of the CliniPAK for diagnosis did impacted positively on the diagnosis accuracy of health workers in the state. Prior to the introduction of the CliniPAK, a World bank report had shown that "the diagnostic and therapeutic accuracy	2

	of our workers at the PHC level was a bit low, at about 16 to 17 percent. But with the introduction of the CliniPAK, the diagnostic and therapeutic accuracy scaled up. According to Anita *"I will say that clinic pack in a way had helped, while it lasted, helped to improve and aid the diagnostic accuracy. You know because it would guide them, questions that they don't really have in their mind to ask, it would prompt them to ask and it would lead them to the diagnosis".* It also positively affected the quality of data that were coming from the facilities" However, since the CliniPAK has been discontinued for about 12 months, Anita believed that "the quality of data in those facilities that were using it before might have been affected a bit" even though the initial validation exercise was still ongoing. But Anita was sure that *"for those facilities, there might have been the errors that they were trying to eliminate before might have been introduced".* Those facilities would have loved to continue, but according to her, *"they don't have money for internet service. So that is why I can say that even if they input their data in it, it stays with them, it doesn't go anywhere because of the challenges".*	
6.	Mr. Cole is a coordinator of Primary Health in one of the local government areas in Ondo State. As the primary health coordinator, he oversee health facilities and also conduct clinical services including immunization, family planning, malaria programs and maternal and child health. Mr. Cole has been engaged with the sector for more than 10 years. He shares his experience on the IPP Nigeria project during the phase it was implemented and what he considers as the most significant changes 12 months after the project has ended.<br><br>Mr. Cole described the advantage of the use of CliniPAK how it has affected the quality of data health workers were generating and transmitting. He emphasised that he gets positive reports from health workers on how the CliniPAK has helped them to manage their data. According to him *"Ha, yes, the woman that was there before I left actually told me that "oga this thing is actually helping us here",* you understand what I mean, she said that *"the CliniPAK is helping me". But despite the advantage inherent in the use of the CliniPAK, it is no longer in use in the health facility for long due to project close out and lack of technical maintenance. On the effect this could have on health care delivery, Mr. Cole retorted, "*honestly, they can suffer a relapse because when they are using something and you are getting a lot of improvement with it, in terms of service delivery and data, and you suddenly withdraw it, it may affect negatively. But if it can be improved upon like I have said earlier that if there is a way they can make up for all those site that are no longer functional it will make a lot of great impact and it will improve health service delivery generally.	1
7.	The story of most significant change is told by Mrs. Aba who was the deputy primary health coordinator of Idanre. As the Deputy PHC Coordinator, she was in charge of providing oversight to all health workers in the health facilities and making sure that health activities were implemented as planned. Mrs. Aba was emphatic that the CliniPAK positively impacted on quality and standard of care in the facilities where it was being used. According to her, before the introduction of CliniPAK you need to write and re-write diagnosis and prescription but when CliniPAK was introduced	1

	there was no need *"writing of names again, because the diseases are all written there, you just click on the diseases. Then the drugs are written there, you just click on the drugs you give. So it makes it easier"* Being able to use the CliniPAK to diagnose and prescribe was a significant change. It also enabled health workers to make reference easily with just a click on the case. But since the CliniPAK have been discontinued, according to her, *"the work has not been easy because, you know, we will be looking for the cases one by one, whereas while using CliniPAK, "you will just click on it, and you know, the date is there already and you will just click on it and it will display everything"*. Now without the CliniPAK Mrs. Aba is not happy because, according to her *"the work is more stressful"*	
8	Mrs. Buba is a 25 years old service user and mother of 2 children who have been using the facility for more than one year. She tells her significant story on how the use of VTR and the CliniPAK has impacted on health care.<br><br>Mrs Buba stated that the use of the VTR device has been of significant help to her because they learn a lot from watching some of the videos on the device. According to her, *"On clinic days when we come and we finish singing and exercise they will tell us to watch the pregnant women, the type of food they dish for them and how they give themselves rest or if they are having pains in their tummy, how they quickly rush down to the clinic"*. Mrs. Buba recounted how she had benefited from watching video because it has helped her to gain a lot about things she did not know previously especially about hygiene and eating good food. In her words, "*You know we are in the village and we act like village people, when we watch it we see how we can change, like how we can take care of our home even if we are using one room, if we are using it in a dirty manner we should change because of our health"* She further mentioned how it has positively changed her pattern of diet. *"And the food we eat, because at times we eat only fufu, but they let us know that we are not meant to eat just fufu"*. However, she confirmed that the last time she saw the device being use in the facility was when she came to immunize her baby about 6 months ago. She doesn't know whether the device is still being used anymore or not.	1
